# Allogeneic and xenogeneic lymphoid reconstitution in a *RAG2*^−/−^*IL2RG*^*y*/−^ severe combined immunodeficient pig: A preclinical model for intrauterine hematopoietic transplantation

**DOI:** 10.3389/fvets.2022.965316

**Published:** 2022-10-14

**Authors:** Renan B. Sper, Jessica Proctor, Odessa Lascina, Ling Guo, Kathryn Polkoff, Tobias Kaeser, Sean Simpson, Luke Borst, Katherine Gleason, Xia Zhang, Bruce Collins, Yanet Murphy, Jeffrey L. Platt, Jorge A. Piedrahita

**Affiliations:** ^1^Comparative Medicine Institute, North Carolina State University, Raleigh, NC, United States; ^2^Department of Molecular Biomedical Sciences, College of Veterinary Medicine, North Carolina State University, Raleigh, NC, United States; ^3^Department of Population Health and Pathobiology, College of Veterinary Medicine, North Carolina State University, Raleigh, NC, United States; ^4^Department of Surgery and Microbiology and Immunology, University of Michigan Health System, Ann Arbor, MI, United States

**Keywords:** severe combined immunodeficiency, pig, xenotransplantation, allotransplantation, hematopoietic, RAG2, IL2RG, transgenic

## Abstract

Mice with severe combined immunodeficiency are commonly used as hosts of human cells. Size, longevity, and physiology, however, limit the extent to which immunodeficient mice can model human systems. To address these limitations, we generated *RAG2*^−/−^ *IL2RG*^*y*/−^ immunodeficient pigs and demonstrate successful engraftment of SLA mismatched allogeneic D42 fetal liver cells, tagged with pH2B-eGFP, and human CD34^+^ hematopoietic stem cells after *in utero* cell transplantation. Following intrauterine injection at day 42–45 of gestation, fetuses were allowed to gestate to term and analyzed postnatally for the presence of pig (allogeneic) and human (xenogeneic) B cells, *T*-cells and NK cells in peripheral blood and other lymphoid tissues. Engraftment of allogeneic hematopoietic cells was detected based on co-expression of pH2B-eGFP and various markers of differentiation. Analysis of spleen revealed robust generation and engraftment of pH2B-eGFP mature B cells (and IgH recombination) and mature *T*-cells *(*and *TCR-*β recombination), T helper (CD3^+^CD4^+^) and T cytotoxic (CD3^+^CD8^+^) cells. The thymus revealed engraftment of pH2B-eGFP double negative precursors (CD4^−^CD8^−^) as well as double positive (CD4^+^, CD8^+^) precursors and single positive *T*-cells. After intrauterine administration of human CD34^+^ hematopoietic stem cells, analysis of peripheral blood and lymphoid tissues revealed the presence of human *T*-cells (CD3^+^CD4^+^ and CD3^+^CD8^+^) but no detectable B cells or NK cells. The frequency of human CD45^+^ cells in the circulation decreased rapidly and were undetectable within 2 weeks of age. The frequency of human CD45^+^ cells in the spleen also decreased rapidly, becoming undetectable at 3 weeks. In contrast, human CD45^+^CD3^+^ *T*-cells comprised >70% of cells in the pig thymus at birth and persisted at the same frequency at 3 weeks. Most human CD3^+^ cells in the pig's thymus expressed CD4 or CD8, but few cells were double positive (CD4^+^ CD8^+^). In addition, human CD3^+^ cells in the pig thymus contained human *T*-cell excision circles (TREC), suggesting *de novo* development. Our data shows that the pig thymus provides a microenvironment conducive to engraftment, survival and development of human *T*-cells and provide evidence that the developing *T*-cell compartment can be populated to a significant extent by human cells in large animals.

## Introduction

Severe combined immunodeficient (SCID) mouse models have allowed researchers to answer fundamental questions related to immune system development and function and have served as crucial bridging models in the field of stem cell transplantation, leading to new discoveries with potential pre-clinical application in human medicine (1–3). Commonly used SCID mice models include non-obese diabetic/SCID mice, NODShi.Cg*-Prkdc*^*SCID*^*Il2RG*^*tm*1*Sug*^ and C;129S4*RAG-2*^*tm*1*Flv*^*IL2RG*^*tm*1*Flv*^ (4). All these models harbor multiple genetic modifications abolishing adaptive immunity development, facilitating engraftment of human cells of different lineages. Of these genes, *RAG1, RAG2*, and *ILR2RG*, play a key role in *T*-cell, B cell and Natural Killer (NK) cell development and/or survival. The RAG1 and RAG2 proteins catalyze recombination of B cell receptor (BCR) and *T*-cell receptor (TCR) genes, and inactivation of *RAG1* or *RAG2* leads to a lack of mature B cells and *T*-cells (5). Inactivation of *RAG1* or *RAG2* leads to severe combined immunodeficiency (SCID). *IL2RG*, located on the X chromosome, encodes a subunit of the IL-2 receptor, also present in the IL-4, IL-7, IL-9, IL-15, and IL-21 receptors (6). The result of *IL2RG* inactivation is broadly impaired cytokine signaling, leading to aberrant *T*-cell thymic development, lack or reduced thymus and peripheral lymph node development, and lack of IL-15 NK cell-mediated differentiation (7). Combined, these *IL2RG*^−/−^ characteristics lead to XSCID syndrome (8). In mice inactivation of *IL2RG* and *RAG1* or *RAG2* leads to profound immunodeficiency and allows engraftment of human hematopoietic stem cells (3). Of note, other SCID syndromes may be caused by adenosine deaminase (ADA) deficiency, or Janus kinase 3 (Jak3) deficiency (9).

Despite the fundamental role of SCID mice models, certain limitations exist such as species-specific differences at the anatomical and physiological scale. Pigs resemble humans in terms of organ size, life span, anatomical, and physiological characteristics. Thus, they are considered as important laboratory animal models for biomedical research, especially for tissue engineering and human organ transplantation involving xenograft procedures (10). SCID pigs can be potentially important research tools to facilitate long-term follow-up studies of immune responses, xenotransplantation, stem cells, and cancer over clinically relevant time frames. Furthermore, SCID pigs can play a key role in assessing the safety of stem cell therapies, or the effects of surgery and radiation therapy in transplanted tumors, thus constituting an important preclinical model. Within the last decade there have been numerous SCID pig models created, including *IL2RG* knockout (11–15), *RAG1* and or *RAG2* knockout (16–19), as well as double gene knockouts of *IL2RG, RAG*, or *ARTEMIS* (20–22), resulting in a range of translational research models, including models of infection with human norovirus (20), human iPSC engraftment (17), tumor melanoma cell engraftment (11), and allogeneic hematopoietic lineage engraftment (15, 17). Only one group to date has shown human *T*-cell restricted xenogeneic engraftment in a *IL2RG*/*ART* null SCID pig (22). Our results confirm and extend those observations using a different double mutant genetic background, the *RAG2*^−/−^*IL2RG*^*y*/−^. From heretofore, we will refer to this line as the *DKO* line. Using the *DKO* line we demonstrate significant allogeneic and xenogeneic engraftment following *in utero* transplantation of pig H2B-GFP tagged fetal liver cells or human CD34^+^ cells, respectively.

## Materials and methods

### Ethics statement

This study was carried out in strict accordance with the recommendations in the Guide for the Care and Use of Laboratory Animals of the National Institutes of Health. The animals used in this study were obtained from a university-owned herd, and all animal procedures were approved by the Institutional Animal Care and Use Committee of North Carolina State University (Raleigh, NC). Animals were sacrificed by one of two methods, intravenous injection of sodium pentobarbital, or penetrating captive bolt euthanasia followed by jugular exsanguination. Both methods meet the recommended guidelines of the American Veterinary Medical Association for euthanasia in pigs. All surgeries were performed under isoflurane anesthesia, and a post-surgical regimen of bupivacaine, Banamine-S was administered to minimize pain. Xenotransplantation procedure and management of xenografted pigs was performed at an approved facility within the College of Veterinary Medicine under proper guidelines in accordance with IACUC protocols. All human samples were obtained from healthy donors under an approved IRB to the University of Michigan and shipped to North Carolina State University without any identifying information.

### Generation of *DKO* porcine fetal fibroblast line

**Figure 1:**
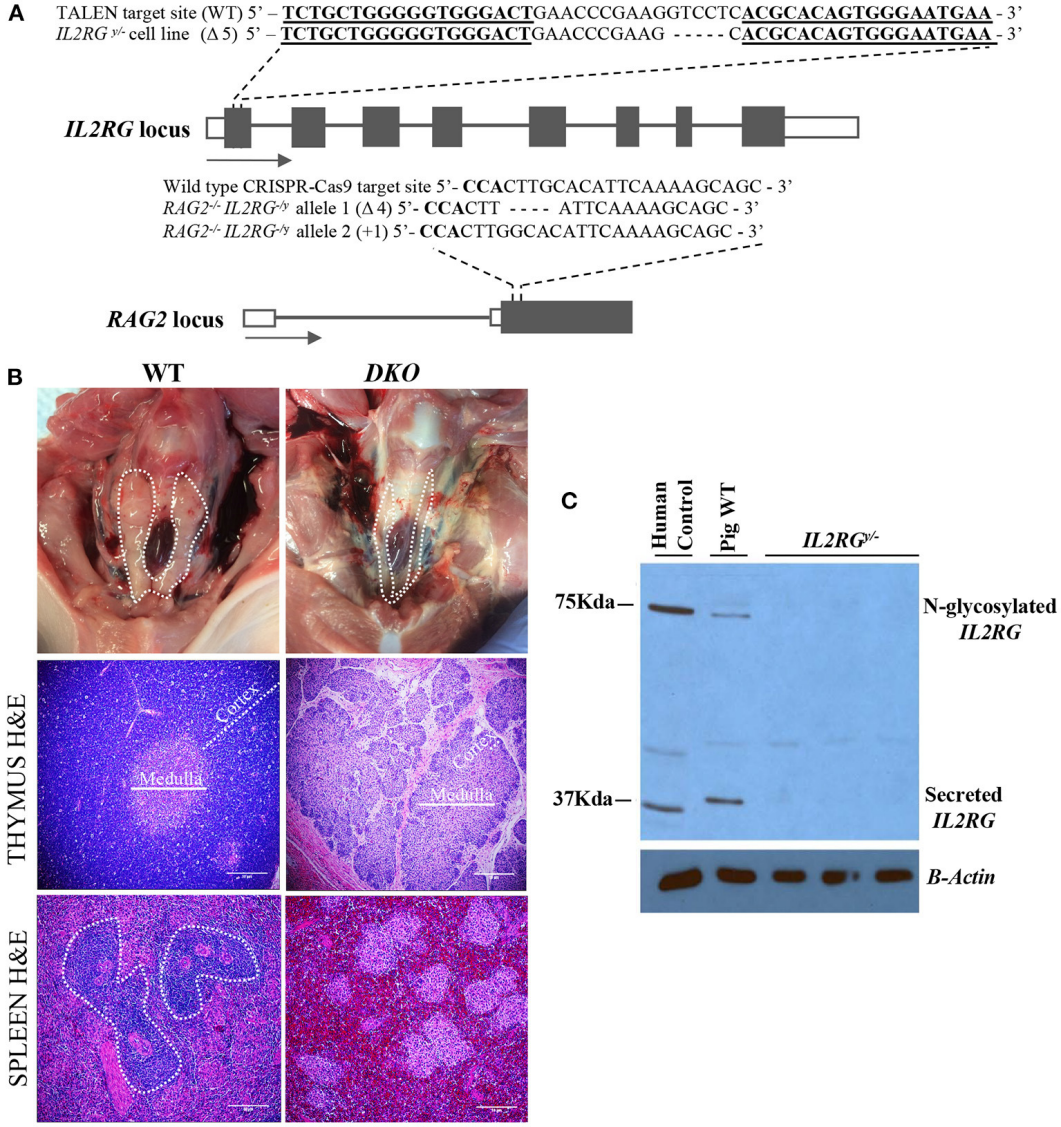
Genotype and initial characterization of *DKO* pigs. **(A)** DNA sequencing of the *IL2RG* and *RAG2* locus target regions detected from *DKO* pigs. Wild type (WT) sequence is shown as a reference. TALEN binding sites are represented by bold and underlined sequences, while bold sequences represent the PAM sequence. **(B)** Thymus (cervical region) from wild-type pigs could be easily visualized (dotted contour white line) while in age-matched *DKO* pigs it was nearly undetectable. H&E microscopic (20x magnification) evaluation of the *DKO* thymus revealed a poorly developed thymus lacking proper cortex (white dotted line), Medulla is also indicated (solid white line). The spleen of wild type age-matched pigs had visible germinal centers (dotted contour white line) while the spleen of *DKO* pigs did not. **(C)** Western blot for *IL2RG* using wild type (WT) and mutant pig heart tissue lysates obtained from *IL2RG*^*y*/−^ pig fetuses. Human peripheral blood served as human positive control.

All gene edits were carried out in fetal fibroblasts obtained from D30 fetuses collected from three-way Yorkshire × Landrace × Duroc crossbred animals from the Swine Educational Unit at North Carolina State University as previously described (23). Initially, an *IL2RG*^*y*/−^ fetal fibroblast line was generated using TALENs (Supplementary Figures 1A,B). This cell line was selected for further targeting of the *RAG2* locus *via* CRISPR-Cas9 using custom-designed *gRNAs* targeting exon 2 and a hygromycin reporter plasmid containing the *RAG2* CRISPR-Cas9 target sequence (Supplementary Figures 1C–E). This reporter plasmid consists of a red fluorescent protein (RFP) gene as a transfection control, and an out-of-frame hygromycin gene fused to GFP. Gene editing after cleavage of the *RAG2* target results in correction of the out-of-frame fusion hygromycin and GFP gene allowing for both hygromycin selection using 2 mg/ml for 48 h, or and separation of cell by FACS based on GFP expression to enrich for *RAG2* mutants. Following hygromycin and GFP selection/enrichment, single cell clonal colonies were generated by seeding at limiting dilutions, and screening performed by PCR followed restriction enzyme digestion using a *HpyCH4V* restriction site overlapping the gRNA target site (Supplementary Figure 1F). Genomic DNA from single cell colonies were isolated, primers designed to amplify a 190 bp amplicon containing the restriction site, followed by *HpyCH4V* digestion and DNA sequencing of the *RAG2* locus target site. A colony containing biallelic mutations of one bp insertion in one allele, and a four base pair deletion in the other allele, was used to generate the *DKO* transgenic pigs *via* SCNT (Figure 1A).

### Generation of *DKO* pigs *via* somatic cell nuclear transfer

*DKO* pregnancies were generated *via* SCNT as previously described (24). All chemicals were purchased from Sigma (St. Louis, MO) unless otherwise specified. Oocytes from mixed commercial breed sows were collected from local slaughterhouses. Cumulus cells were removed from the oocyte by vortex for 5 min in 0.1% bovine testicular hyaluronidase. Oocytes were incubated in manipulation media (Ca-free NCSU-23 with 5% FBS) containing 5 μg/mL bisbenzimide and 7.5 μg/mL cytochalasin B for 5 min. Following the incubation period, oocytes were enucleated by removing the first polar body and metaphase II plate. Single cells were injected and fused to each enucleated oocyte. Fusion/activation was induced by two DC pulses of 140V for 40 μsec in 280 mM mannitol, 0.001 mM CaCl_2_, and 0.05 mM MgCl_2_. After fusion/activation, oocytes were placed back in NCSU-23 medium with 0.4% BSA and cultured at 38.5°C, 5% CO_2_ in a humidified atmosphere for less than an hour, before being surgically transferred into a synchronized recipient.

### Genotyping of *DKO* Double Knock-out (*DKO*) Pigs

For the *IL2RG* genotyping gDNA was isolated from porcine fetal fibroblast colonies and generated pigs and the primers 5′ CCACTGGAGTTTTTCATTTTGATG 3′ and 5′ ATCCGAAAGCTCATTATTTGGTGT 3′ used to amplify the TALEN binding site and flanking region (~1 Kb) under the following conditions 98°C for 1 min; 35 cycles (98°C for 10 s, 64.5°C for 10 s, 72°C for 15 s), 72°C for 1 min. Restriction digest with *AvaII* was performed to detect mono allelic indels of the *IL2RG* locus. For *RAG2* genotyping gDNA was isolated from porcine fetal fibroblast colonies and generated pigs and the primers 5′ CCACTGGAGTTTTTCATTTTGATG 3′ and 5′ ATCCGAAAGCTCATTATTTGGTGT 3′ used to amplify the CRISPR-Cas9 binding site and flanking region (190 bp) under the following conditions 98°C for 1 min; 35 cycles (98°C for 10 s, 64.5°C for 10 s, 72°C for 10 s), 72°C for 1 min. Restriction digest with *HypCH4V* was performed to detect mono and biallelic indels of the *RAG2* locus.

### Allogeneic transfer donors: Swine Leukocyte Antigen (SLA) Matching of PH2B-EGFP donors and *DKO* recipient cell line and isolation of liver hematopoietic stem cells

To ensure that the allogeneic transplants were done across different swine leukocyte antigen complex (SLA) haplotypes, both the pH2B-eGFP D42 fetal liver donor cells derived from a gene edited line we previously generated that contains the pH2B-GFP under the ACTB promoter (25), and the recipient *DKO* line were typed *via* a previously described PCR assay using site specific primers (PCR-ssp) (26–28). The assay included primers specific for identification of pig MHC-I pig loci *SLA-1, SLA-2* and *SLA-3*, and MHC-II pig loci *DRB1, DQB1* and *DQA* (Supplementary Figures 2A,B), therefore testing the pig *SLA* analogues to classical *HLA* regulating the success of hematopoietic stem cell transplantation.

Allogeneic hematopoietic stem cells were obtained from fetal livers. Midgestational fetal liver cells are known to be enriched for hematopoietic stem cells and are routinely used in mouse models of *in utero* hematopoietic stem cell transplantation (29, 30). Forty-two days pH2B-eGFP fetal liver cells were isolated using a previous reported protocol (31). Briefly, D42 pH2B-eGFP fetal male livers (3 separate donors) were minced, digested in liver digestion media (Gibco #17703034), and washed with Iscove Modified Dulbecco's Media (Gibco #12440053). Fetal liver parenchymal and hematopoietic stem cells were separated by density centrifugation and the cell fraction enriched for hematopoietic stem cells cryopreserved in Hanks' Balanced Salt Solution (HBSS) containing 5% DMSO, 0.3 M Sucrose and no calcium/magnesium.

### Xenogenetic transfer donors: Immunomagnetic selection of CD34 ^+^ cells

Peripheral blood mobilized stem cells (PBMC) from two unrelated male adult donors were utilized as the source of stem cells. Hematopoietic stem cell mobilization was achieved by conducting standard mobilization treatment with G-CSF, followed by peripheral blood harvest and cryopreservation. Each cryopreserved bag contained ~50 ml per bag. A total of four bags were used per pregnancy. Frozen peripheral blood containing mobilized hematopoietic stem cells were thawed and washed in autoMACS Rinsing Solution (Miltenyibiotec #130-091-222) containing MACS BSA Stock Solution (#130-091-376) at 1:20 dilution. Mononuclear cells were isolated *via* centrifugation in Ficoll-Paque PLUS (GE Healthcare #71-7167-00 AG), yielding ~600 million PBMCs containing mobilized HSCs. Mononuclear cells were incubated with CD34 MicroBead Kit—UltraPure (Miltenyibiotec #130-100-453), and CD34^+^ human cells isolated with a magnetic system described previously. Purification to 97.7% was confirmed *via* flow cytometry (Supplementary Figure 2C). Sexing of donor cells were done by PCR using the X linked ALT1 gene (5′- CCCTGATGAAGAACTTGTATCTC-−3′ and 5′- GAAATTACACACATAGGTGGCACT- 3′), and the human SRY from the Y chromosome (5′-CATGAACGCATTCATCGTGTGGTC-3′ and 5′-CTGCGGGAAGCAAACTGCAATTCTT-3′). PCR conditions were 98°C for 1 min; 35 cycles (98°C for 10 s, 58°C for 10 s, 72°C for 8 s), 72°C for 1 min (Supplementary Figure 2D).

### In utero hematopoietic transplantation of PH2B-EGFP pig fetal liver hematopoietic stem cells or CD34 ^+^ cell transplantation

Allo and Xenotransplantation procedures and management of allografted and xenografted pigs was performed at an approved facility within the College of Veterinary Medicine under proper guidelines in concordance with IACUC protocols. At D42 of gestation a wildtype sow carrying a transgenic *DKO* pregnancy was prepared for surgery. pH2B-eGFP fetal liver cells were thawed and ran through a live dead magnetic isolation system (Miltenyibiotec #130-090-101) using LS Columns (Miltenyibiotec #130-042-401) placed in a QuadroMACS Separator (Miltenyibiotec #130-090-976). In utero transfer were carried out essentially as described (22) with the exception that the cells were injected intra-hepatically, not intraperitoneally, *via* ultrasound guided (Aloka) procedure, using a PAN chiba (25 gauge × 6 cm) cytological aspirating needle (Gallini Medical Devices ref #PA25-6). First, each fetus was located with the ultrasound and scanned from cranial to caudal direction until the hyperechoic liver structure and central vein (hypoechoic) was identified caudally to the thoracic cavity. The probe was held in position and the PAN chiba needle inserted through the uterine wall until it could be identified in the ultrasound. One uterine horn was scanned at a time, from the tip of the uterine horn (next to the ovary) all the way down to the uterine horn bifurcation. Per fetus, a total of 30 ul of saline cell suspension was slowly injected. Deposition of cell suspension solution was visible in the ultrasound screen, followed by retraction of the needle while maintaining contact ultrasound visualization of the liver.

For allogeneic engraftment, 3 *DKO* fetuses from one pregnancy received 3.5 × 10^6^ fetal liver hematopoietic stem cells isolated from D42 pH2B-eGFP fetal liver donors (1 unique donor per *DKO* recipient). For xenogeneic engraftment with human hematopoietic stem cells, 7 × 10^6^ viable human CD34^+^ cells/fetus were transplanted *via* intra-hepatic ultrasound-guided fetal injection into *DKO* D42 fetuses. Fetuses from two pregnant gilts were injected with cells from two independent male human donors and resulted in three and four viable pigs at term (total n = 7 viable term pigs). Non-injected *RAG2*^−/−^*IL2RG*^*y*/−^ (*n* = 3) and injected wild type *RAG-2*^+/+^*IL2RG*^+/*y*^ (*n* = 2) served as controls. (CD34^+^ enrichment and donor sexing data are presented in (Supplementary Figures 2C,D). A timeline schematic of all *in vivo* procedures and sample harvests performed is provided in Supplementary Figure 3.

### Histological and immunofluorescence analyses

*DKO* and aged matched wild type controls pigs were euthanized, and lymphoid organs isolated. Age of euthanasia ranged from 1 day to 3 weeks and are stated in each figure legend. For hematoxylin and eosin (H&E) and immunohistochemistry staining, the thymus and spleen were fixed in 10% neutral buffer formalin for 24 h, followed by 70% ethanol for 24 h. For immunofluorescence analysis, fresh tissue samples were placed in 30% sucrose at 5°C overnight, followed by OCT embedding and freezing. For IHC analysis of paraffin embedded tissues of non-injected *DKO* pigs, samples were stained with mouse anti-human CD79α unconjugated (BioRad/AbDSerotec #MAC2538), anti-human CD3 (Dako #A0452), and rabbit anti-human CD335 (BIOSS #bs-10027R). For detection of human cells fresh OCT embedded thymus and spleen were immunostained with mouse anti-human CD45 FITC labeled (BD #555482). Cell numbers were obtained *via* ImageJ processing and analysis and expressed as the number of cells/higher power field (40x magnification). At least three images were acquired and analyzed.

### Flow cytometry

Peripheral blood (PB) collected into EDTA containing tubes and single-cell suspension from thymus and spleen from *DKO* pigs were processed and prepared for antibody staining immediately after collection. A total of 1 × 10^6^ cells were stained per sample. For detection of pig cells, we used the following antibodies. For *T*-cells, mouse anti pig CD3 epsilon Alexa Fluor 405 labeled (Novus #NBP1-28225AF405), mouse anti pig CD8 PE labeled (BD #559584) and mouse anti pig CD4 PE-Cy7 labeled (BD #561473). For detection of pig B cells mouse anti human CD79α Alexa Fluor 647 Labeled (BioRad/AbDSerotec #MCA2538A647) using LEUCOPERM kit (BioRad/AbDSerotec #BUF09) for intracellular staining. For detection of pig NK cells, mouse anti pig CD335 (BioRad/AbDSerotec #MCA5972GA) conjugated with APC-Cy7 with the kit LN#131PACCY7 (BioRad/AbDSerotec). Samples were incubated for 1h at 5°C in staining buffer (BD #554657), followed by red blood lysis and fixation with lysing solution (BD #349202) and washing. All flow data was gated and displayed as a percentage of gated mononuclear cells. For the detection of human lymphocytes from peripheral blood, thymus, spleen of xenografted pigs were stained with mouse anti-human CD45-PE-CF594 (BD #562279), for *T*-cell detection, mouse anti-human CD3 PE labeled (BD #555333), mouse anti-human CD8 APC labeled (BD #340584) and mouse anti-human CD4 FITC labeled (BD #561842) were used. For detection of human B cells, mouse anti-human CD19 FITC labeled (BD #564456), for human NK cells mouse anti-human CD335 FITC labeled (BD #564536). We conducted a series of flow cytometry antibody cross reactivity studies by mixing human and pig hematopoietic cells, showing no cross reactivity, as well as staining non-injected *DKO* pigs as negative controls. Flow cytometry analysis was performed with Flow Jow software. Gating strategy was applied to remove dead cells and debris, followed by a single cell gate, followed by a mononuclear cell gate and analysis of the markers of cells of interest. *T*-cell subsets were analyzed by gating of CD3^+^ cells.

### PCR assay for detection of pig V(D)J rearranged IgH and TCR-β locus

Genomic DNA was isolated from peripheral blood mononuclear cells, thymus and spleen. The previous reported primers (32, 33) D1J1-F and D1J1-R were used it to identify D-J rearrangement of the *TCR-*β locus in peripheral blood and thymus of *DKO* pigs with Phire Hot Start II DNA Polymerase (Thermofisher #F122L) under the following conditions, 98°C for 1 min; 35 cycles (98°C for 10 s, 68°C for 10 s, 72°C for 30 s), 72°C for 1 min. *IgH* rearrangement was detected by PCR utilizing the previous reported primers FR1 and JH (rearranged *IgH*) and D4-F and J3-R (germinal control) (18) with Phire Hot Start II DNA Polymerase under the following conditions 98°C for 1 min; 35 cycles (98°C for 10 s, 62.5°C for 10 s, 72°C for 30 s), 72°C for 1 min.

### PCR assay for detection of VαJα human TREC recombination

To demonstrate human TCR rearrangement in the thymus of xenografted pigs, previous reported primers (32, 33) were used to amplify the δ*Rec-*Ψ*J*α segment of the human coding joint *T*-cell receptor excision circle (*V*α*J*α TREC). This extrachromosomal DNA circle indicates proper δ*Rec-*Ψ*J*α recombination. Amplification of the *IL2RG* locus was used as control for DNA loading. Ten ng of gDNA from thymus and blood were used as templates, thymus of a *DKO* non-injected pig served as negative control, while gDNA from donor cells used for xenoengraftment was used as positive control. Forward and reverse primers were 5′ CTAATAATAAGATCCTCAAGGGTCGAGACTGTC 3′ and 5′ CCTGTTTGTTAAGGCACATTAGAATCTCTCACTG 3′. PCR conditions for *V*α*J*α TREC were 98°C for 1 min; 35 cycles (98°C for 10 s, 69°C for 10 s, 72°C for 15 s), 72°C for 1 min.

### Statistical analysis

Statistical analysis and graphs were generated in GraphPad Prism 8. Data comparison between two groups was performed *via* two-tailed unpaired t-test with a significance value of 0.05. Multiple group analysis was performed *via* analysis of variance (ANOVA) with Tukey's multiple comparisons test with a significance value of 0.05.

## Results

### Generation of *DKO* gene-edited pigs

The *IL2RG* pig locus was targeted with custom-designed TALENs assembled *via* FLASH system (34). This cell line harbors a 5 base pair deletion of exon 1 of the *IL2RG* locus (Figure 1A). Targeted cells were used for SCNT to generate *IL2RG* null D42 fetuses, and lack of IL2RG protein was confirmed by Western blot (Figure 1C). These fibroblasts were then used to inactivate the *RAG2* gene. A high targeting efficiency, with indel frequency reaching 79% (51% monoallelic and 28% monoallelic) was obtained (Supplementary Figure 1G). Four homozygous and two heterozygous colonies were submitted for DNA sequencing, revealing deletion, point mutations and insertions (Supplementary Figure 4). Sequencing analysis of one colony (#29) revealed biallelic mutations of 4 base pair deletion from one allele and a 1 base pair insertion in the other allele. In silico analysis of both allelic mutations predicted multiple premature stop codons immediately downstream of the CRISPR-Cas9 binding site, leading to lack of RAG2 protein expression. This colony, therefore, was selected as the SCNT donor to generate *DKO* transgenic pigs. A total of 291 (*DKO*) reconstructed embryos were transferred and 2 pregnancies carried to term, generating five *DKO* transgenic pigs that were used for initial analysis, with 1 pig dying at birth resulting in 4 DKO pigs used for phenotype characterization. For xenogeneic engraftment, additional pregnancies were generated, and fetuses injected at D42 of gestation with human CD34 cells.

### Lymphoid lineage analysis of non-engrafted pigs

**Figure 2:**
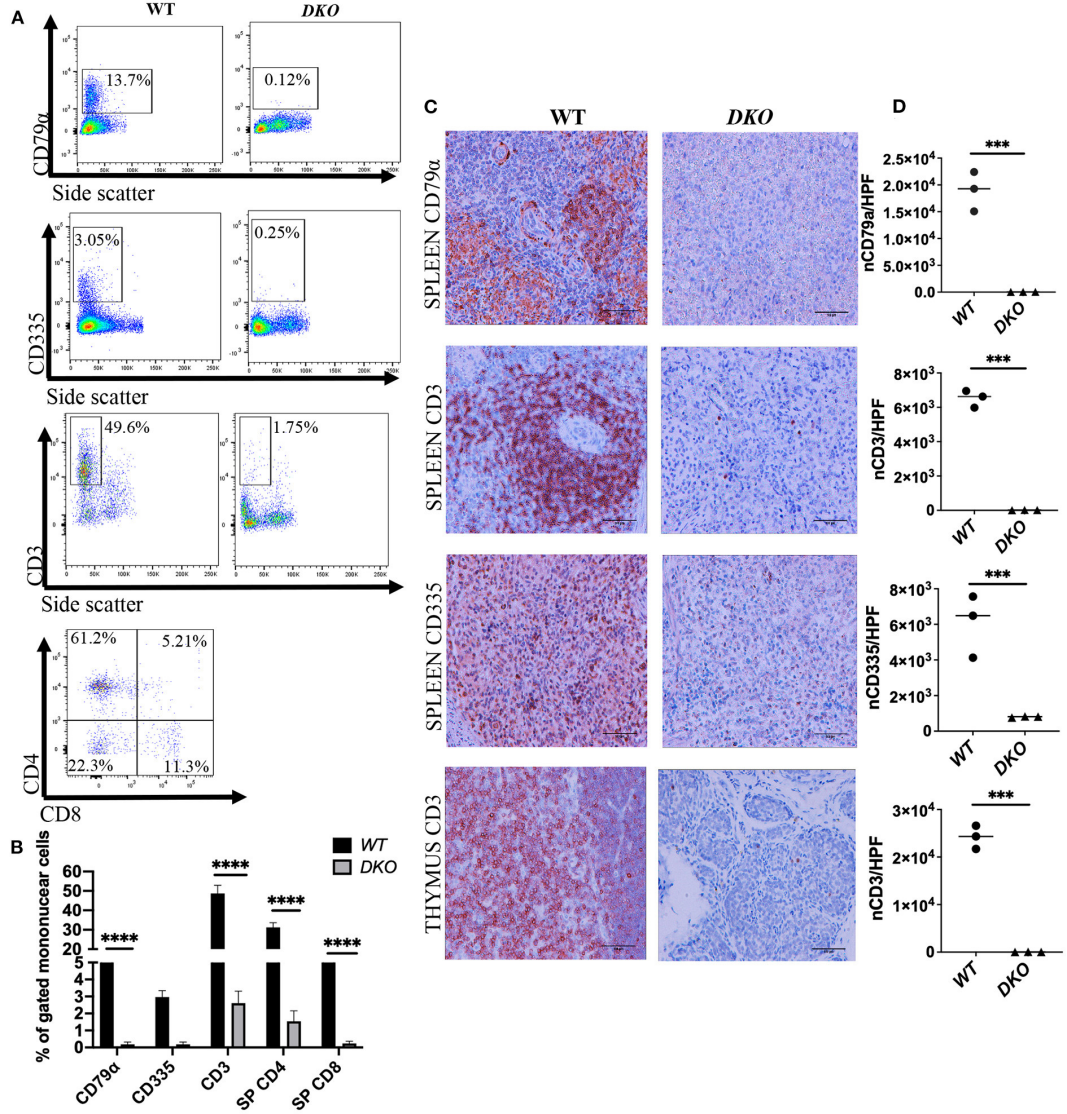
Lymphoid cell analysis of peripheral blood and lymphoid tissue from immunodeficient *DKO* pigs. **(A)** Representative peripheral blood flow cytometry analysis showing CD79α^+^ cells, CD335^+^ cells, CD3^+^ CD4^+^ cells and CD3^+^ CD8^+^ cells for wild type (*n* = 3) and *DKO* (*n* = 4) pigs at 1 week of age. CD79α^+^, CD335^+^, CD3^+^ CD4^+^, and CD3^+^ CD8^+^ are nearly undetectable in the peripheral blood of *DKO* pigs. **(B)** Histogram plot with bar of percentage from gated mononuclear cells for CD79α^+^, CD335^+^, CD3^+^, CD3^+^ CD4^+^ and CD3^+^ CD8^+^ cells. Data represents mean, bars indicate standard deviation, and adjusted *p* values are represented for Tukey's multiple comparisons tests one-way ANOVA with a significance value of 0.05. **(C)** Representative immunohistochemistry (40x magnification) showing spleen staining for CD79α ^+^ (B cells), CD335^+^ (NK cells), and spleen and thymus staining for CD3 (*T*-cells) for wild type (*n* = 3) and *DKO* (*n* = 3) pigs at 3 weeks of age. **(D)** scatter plot with individual values for the number of CD79α^+^ (spleen), CD335^+^ (spleen) and CD3^+^ (thymus and spleen) cells per high power field microscopy. CD79α^+^, CD335^+^, and CD3^+^ cells are nearly absent in the spleen, and CD3^+^ cells are nearly absent in the thymus of *DKO* pigs. Line represents the mean, and adjusted *p* values are represented for unpaired two-sample *t*-test with a significance value of *P* < 0.05. ***P < 0.001, ****P < 0.0001.

Analysis of lymphoid lineage of peripheral blood cells from age-matched *DKO* (*n* = 4) pigs was performed *via* flow cytometry at 1 week of age with antibodies specific for pig CD3, CD4, CD8, CD79α and NKp46 (CD335). *DKO* pigs had a marked reduction (% gated mononuclear cells) of CD79α ^+^ cells compared to controls (*p* < *0.0001*) (Figures 2A,B). Analysis of *T*-cells populations in peripheral blood *via* expression of CD3 ^+^ CD4 ^+^ and CD3 ^+^ CD8 ^+^ revealed that *DKO* pigs had reduced levels of CD3^+^CD4 ^+^ *T*-cells (*p* < *0.0001*) and CD3^+^CD8^+^ *T*-cells (*p* < *0.0001*) (Figures 2A,B). Next, we studied peripheral blood levels of NK cells *via* flow cytometric analysis using the marker NKp46 (CD335) (35). *DKO* pigs had a marked reduction in levels of CD335^+^ cells in peripheral blood (~0.8%), however, this was not significantly different from WT (~2.95%) (*p* = 0.1575) (Figures 2A,B).

Histopathological analysis of the spleen of *DKO* pigs showed was devoid germinal centers (Figure 1B). No macroscopically visible lymph nodes were detected in *DKO* pigs, likely a consequence of *IL2RG* inactivation. Spleen IHC for CD79α showed a marked reduction of CD79α ^+^ cell in *DKO* (*p* = *0.0009*) (Figures 2C,D). Similarly, there was a marked reduction of CD3 ^+^ cell and CD335 in *DKO* pigs (*p* < *0.0001* and *p* = *0.0066*, respectively*)* (Figures 2C,D). The thymus of *DKO* pigs was poorly developed, lacking proper cortical zone development (Figure 1C) and exhibited a marked reduction of CD3^+^ cells (*p* < *0.0001*) (Figures 2C,D). These observations are in concordance with previous reports (20).

Analysis of RAG2 activity using PCR assays to detect molecular *V(D)J IgH* rearranged locus of B cells in the spleen and *D-J TCR-*β in the thymus showed that rearranged loci could be detected in the control animals but not in *DKO* pigs (Supplementary Figure 5). Together these results extend previous phenotypic analysis of *DKO* deficient pigs (20) and confirm that *DKO* pigs display a severe combined immunodeficient phenotype.

### SLA typing of donor cells and host *DKO* line

SLA typing of donor and recipients demonstrated transplants to be bidirectional mismatches (0/8) for donor 1 and 2, with only one allele being shared (*SLA-3 03xx*) and mostly unidirectional mismatch (0/8) for donor 3 with 7 alleles being common shared (*SLA-1 04xx, SLA-2 04xx, SLA-3 03xx, 04xx, DRB1-07xx, DQB1 02xx, 09xx*). Only one perfect haplotype match was observed for *DQA* between donor 3 and recipients. Therefore, all donors were classified as poor hematopoietic stem cells donors (Supplementary Figures 2A,B).

### Allogeneic PH2B-EGFP lymphoid cells in lymphoid tissue of *DKO* allografted pigs

**Figure 3:**
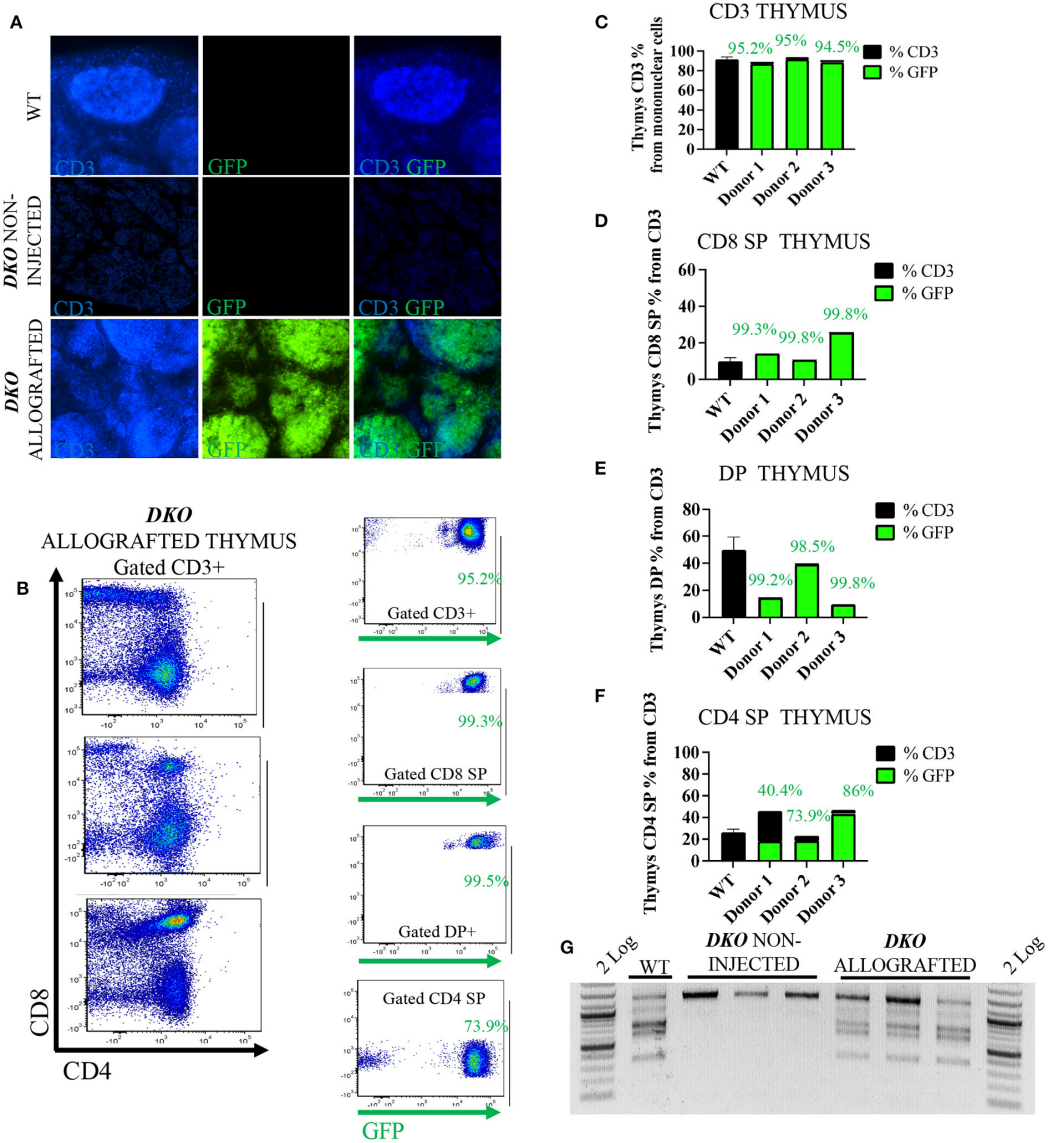
*In utero* allogeneic transplantation of pH2B-eGFP fetal liver cells restored *T*-cells and *TCR-*β recombination in the thymus of 1-week-old *DKO* pigs. **(A)** Immunofluorescence staining (20x magnification) for CD3 (blue), and pH2B-eGFP (GFP-green). Wild-type thymus contains CD3 positive cells, but not pH2B-eGFP (GFP) positive cells. Thymus of non-injected *DKO* pigs is negative for both CD3 and pH2B-eGFP cells. The thymus of allografted *DKO* pigs contains CD3 cells, all overlapping with pH2B-GFP positive cells. **(B)** flow cytometry plots from the thymi of three allografted *DKO* pigs, showing expression of CD4 and CD8 (gated on CD3^+^ cells). Also shown are representative plots of GFP^+^ CD3^+^, CD3^+^CD8^+^, CD3^+^CD4^+^CD8^+^ (DP) and CD3^+^CD4^+^ cells. **(C–F)** Histogram showing thymic percentage and chimerism of CD3^+^ cells **(C)**, CD3^+^CD8^+^ cells **(D)**, DP cells **(E)** and CD3^+^CD4^+^ cells **(F)** from thymi of all three *DKO* allografted pigs, while a wild type (*n* = 3) serves as positive control. The green histogram represents the levels of pH2B-eGFP chimerism, indicating the percentage of allograft donor-origin cells from the respective population. **(G)** PCR assay for the identification of rearranged *TCR*-β locus from thymus gDNA. Non-injected *DKO* pig thymus served as a negative control and a wild type as the positive control. All three allografted pig thymi display rearranged *TCR-*β locus.

**Figure 4:**
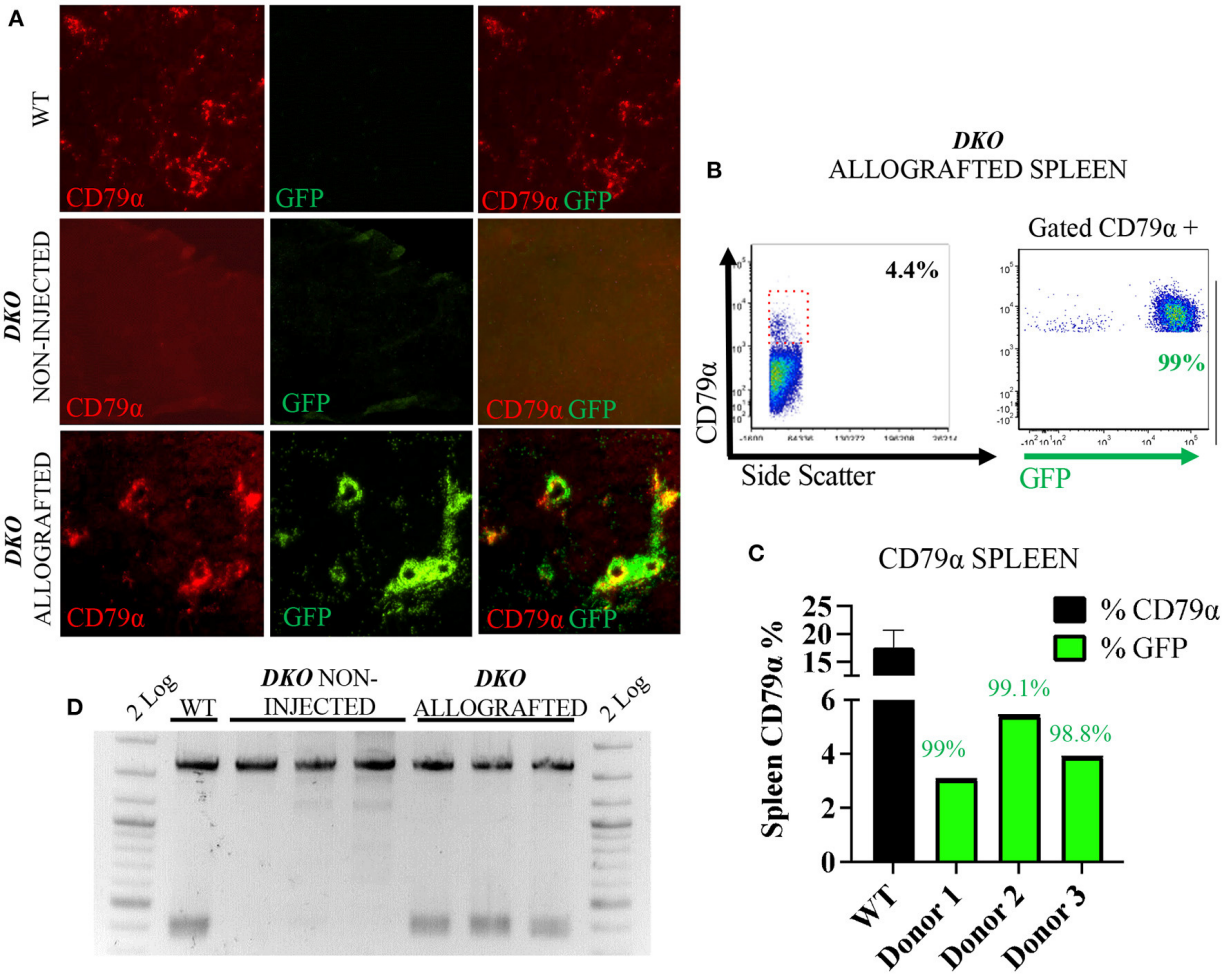
In utero allogeneic transplantation of pH2B-eGFP fetal liver cells restored B cells and *IgH* recombination in the spleen of 1-week-old *DKO* pigs. **(A)** Immunofluorescence staining (20x magnification) for CD79α (red), and pH2B-eGFP (GFP-green). Overlay images are also represented. Wild-type spleen contains CD79α+ cells, but not pH2B-eGFP positive cells. Spleen of non-injected *DKO* pigs is negative for both CD79α+ and pH2B-eGFP cells. The spleen of allografted *DKO* pigs contains CD79α+ cells, all overlapping with pH2B-eGFP cells. **(B)** Representative flow cytometry plot from *DKO* allografted spleen showing expression of CD79α. A red box indicates the CD79α positive cells within the spleen. Representative flow cytometry of gated CD79α+ cells expressing pH2B-eGFP is also shown. **(C)** Histogram showing the percentage of CD79α+ cells from the spleen of all three *DKO* allografted pigs, while wild type (*n* = 3) serves as a positive control. Green histogram represents the levels of pH2B-eGFP chimerism, indicating the percentage of allograft donor-origin cells in the CD79α+ population. **(D)** PCR assay for the identification of rearranged *IgH* locus from spleen gDNA. Non-injected *DKO* spleen serve as negative controls, and a wild type as a positive control. All three allografted piglets display *IgH* rearranged locus.

The pH2B-eGFP positive donor cells could be identified and overlapped with CD3^+^ cells as demonstrated by immunofluorescence analysis of allograted thymus at 1 week of age, while absent from aged matched wild type spleen and non-injected *DKO* pigs (Figure 3A). Additionally, allogeneic *T*-cell engraftment was confirmed *via* flow cytometry detection of pH2B-eGFP and staining the thymus of a *DKO* pig for CD3, CD4 and CD8. The pH2B-eGFP positive cells were easily identified (Figure 3B) and supported identification of engraftment of basic *T*-cell phenotypes in all three *DKO* pigs receiving each a different donor haplotype (Figures 3B–F). In contrast, pH2B-eGFP positive cells were absent from the thymus of aged-matched non-injected *DKO* pigs (Figure 3A). In addition, TCR-β rearrangement was detected in the thymus of allografted pigs (Figure 3G). The pH2B-eGFP positive donor cells could also be identified and overlapped with CD79α^+^ cells in the spleen (Figure 4A). Flow cytometry analysis of allografted *DKO* pigs spleen at 1 week of age showed >90% GFP^+^ CD79α^+^ cells (Figures 4B,C). As expected, *IgH* rearrangement was detected in the spleen of wild type and allografted pigs, while absent in *DKO* pigs (Figure 4D). Together, these results demonstrated that *in utero* allogeneic stem cell transplantation can be used successfully to establish lymphoid allogeneic engraftment in *DKO* pigs.

### Human lymphoid cells in cord and peripheral blood from Xenografted *DKO* pigs

**Figure 5:**
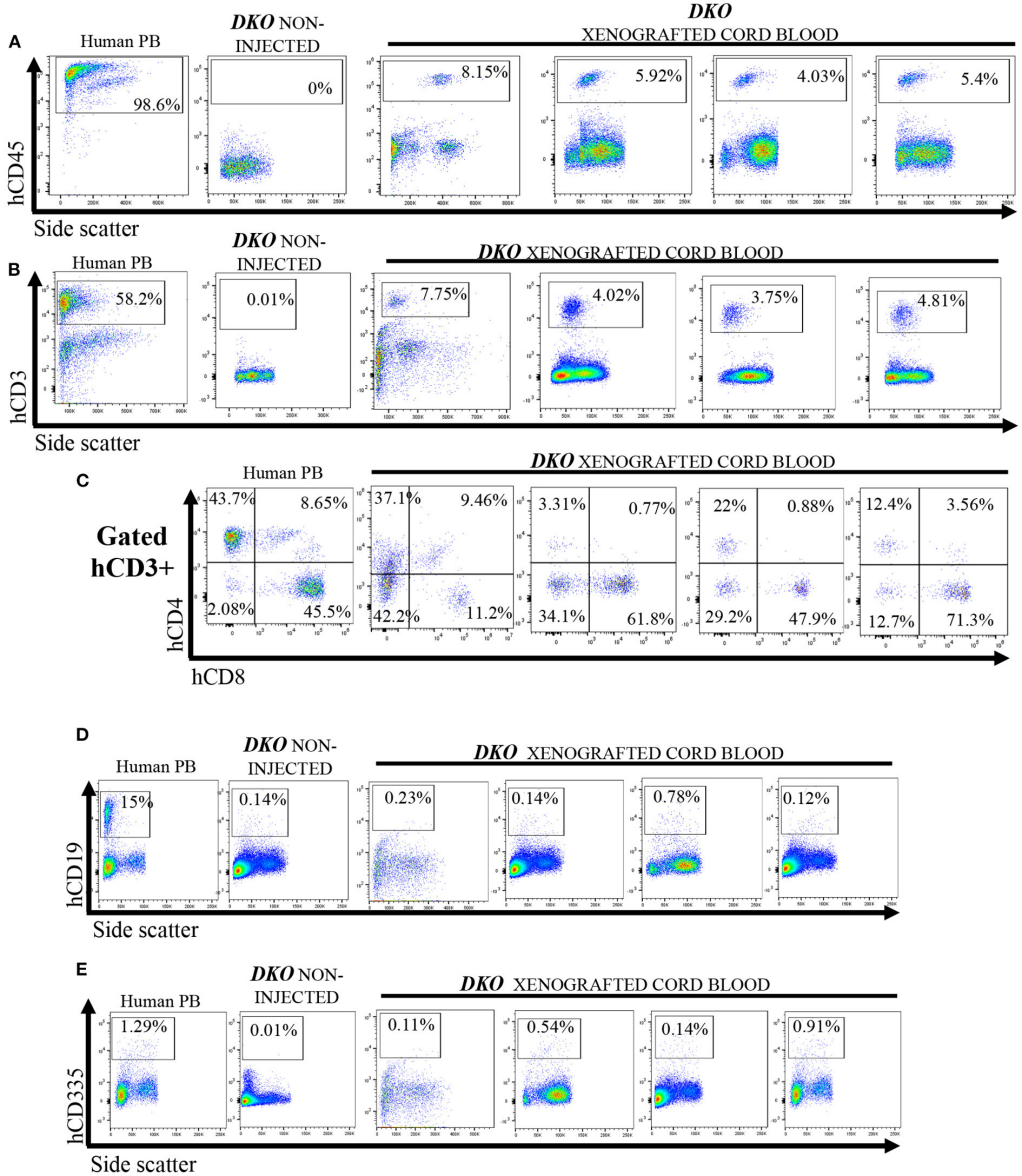
Cord blood flow cytometry analysis for human *T*-cells, B cell and NK cells from non-injected and xenografted *DKO* pigs. Flow cytometry analysis for human lymphoid cells in cord blood from xenografted pigs (*n* = 4). Peripheral human blood served as positive controls, while non-injected *DKO* pigs (*n* = 4) served as negative controls. All xenografted pigs (*n* = 4) showed; **(A)** A distinct hCD45 population of circulating mononuclear cells. **(B)** A hCD3 population of circulating cells with similar percentages as hCD45 cells. **(C)** Expression of hCD4 and hCD8 among hCD3^+^ cells, presence of SP hCD4 and SP hCD8, DN and DP. **(D)** Xenografted *DKO* pigs showed nearly undetectable levels of hCD19 (B-cells) and a small but significant (*P* < 0.05) presence of hCD335 **(E)** cells when compared to non-injected *DKO* pigs. Only one representative non-injected animal and human peripheral blood (PB) sample is shown.

**Figure 6:**
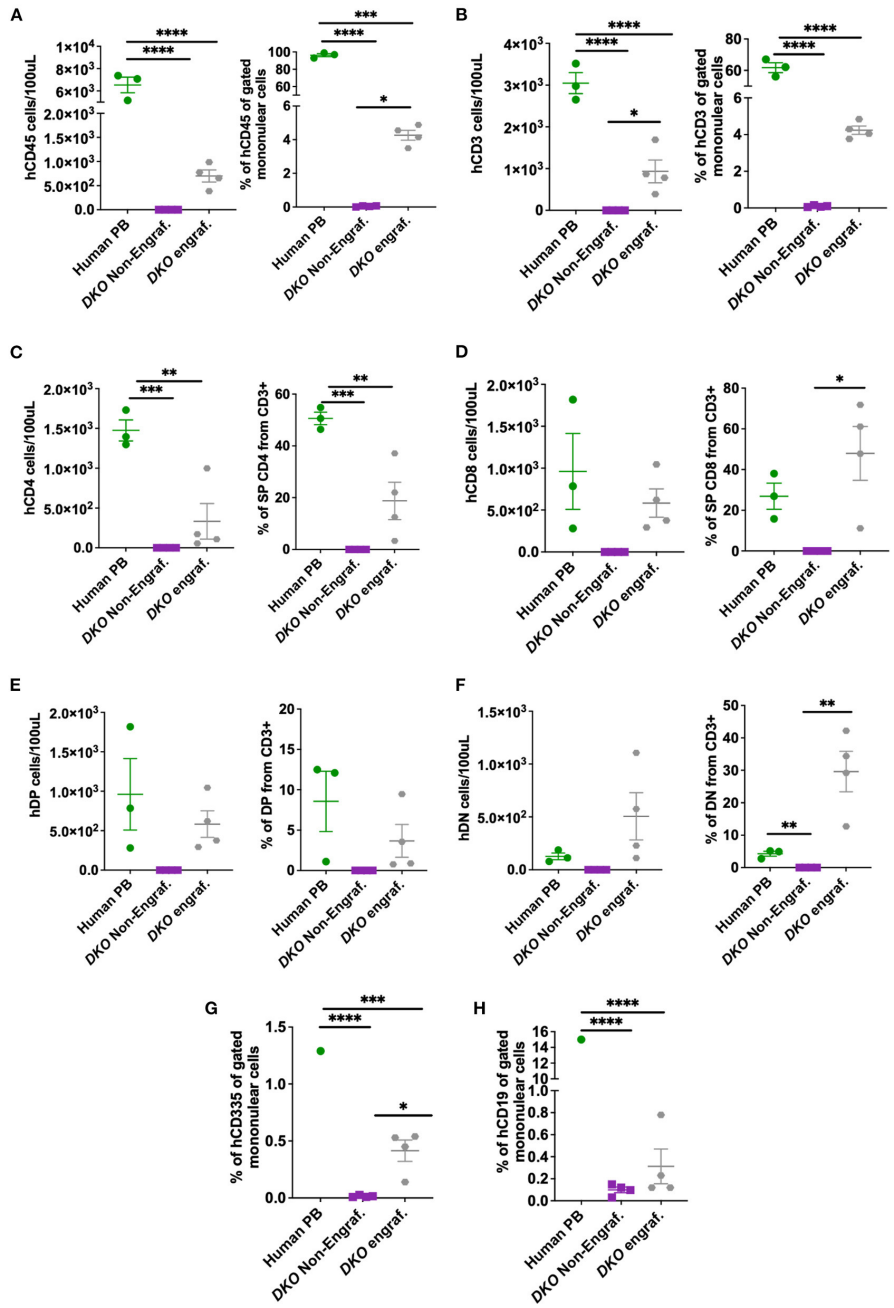
Cell number and percentage quantification of cord blood flow cytometry data from *DKO* xenografted pigs. **(A–F)** Scatter plot showing number of human cells/100 μL and percentage from mononuclear cell (from CD3^+^ for T subsets) or pig cord blood for human CD45, human CD3, human CD4, human CD8, human double positive (hDP) and human double negative (hDN) *T*-cells. Human blood served as positive control while the blood of non-injected *DKO* pigs served as a negative control. The line represents the mean, and adjusted *p* values are represented for Tukey's multiple comparisons tests one-way ANOVA with a significance value of *P* < 0.05. **(G,H)** Scatter plot showing human CD335 and CD19 percentage from mononuclear cells. *P < 0.05, **P < 0.01, ***P < 0.001, ****P < 0.0001.

Next, we investigated if *DKO* pigs could engraft with human hematopoietic stem cells using the same transplantation method with higher total cell dose/fetus. Cord blood was collected from control and xenografted *DKO* pigs at birth and analyzed *via* flow cytometry. Human peripheral blood (*n* = 3) was used as a reference. *DKO* non-injected (*n* = 4) and wild type pigs served as negative controls. Of the seven *DKO* fetuses injected with human hematopoietic stem cells, four had a detectable (>1.0%) population of human CD45^+^ cells at birth, with percentages differing statistically from *DKO* non- injected (*n* = 3) (*p* = *0.0128*) (Figures 5A, 6A). Engraftment ranged from 3 to 4%. Human CD3 staining showed detectable human CD3^+^ cell percentages that did not differ statistically (*p* = *0.14*) from *DKO* non-injected levels, although it differed on cell number/100 ul of blood (*p* = *0.0261)* (Figures 5B, 6B). Phenotypic characterization of human CD3 cells revealed the presence of human double negative, hDP (CD3^+^CD4^+^CD8^+^) and human single positive *T*-cells, (hCD4 SP (CD3^+^CD4^+^CD8-), hCD8 SP (CD3^+^ CD4-CD8^+^) (Figures 5C, 6C–F). When compared to human adult peripheral blood, xenografted pigs had higher percentages of hCD8 SP and hDN *T*-cells compared to adult human peripheral blood (*p* = *0.0103* and *p* = *0.0015*, respectively) (Figures 5C, 6C–F). Interestingly, cord blood of *DKO* pigs showed detectable (different from *DKO* non-engrafted, *p* = *0.0127*) levels of human NK cells (CD335), while having nearly undetectable levels of human B cells (CD19^+^). Taken together, these results suggest *DKO* pigs sustain restricted human *T*-cell lineage engraftment after *in utero* hematopoietic stem cell transplantation.

**Figure 7:**
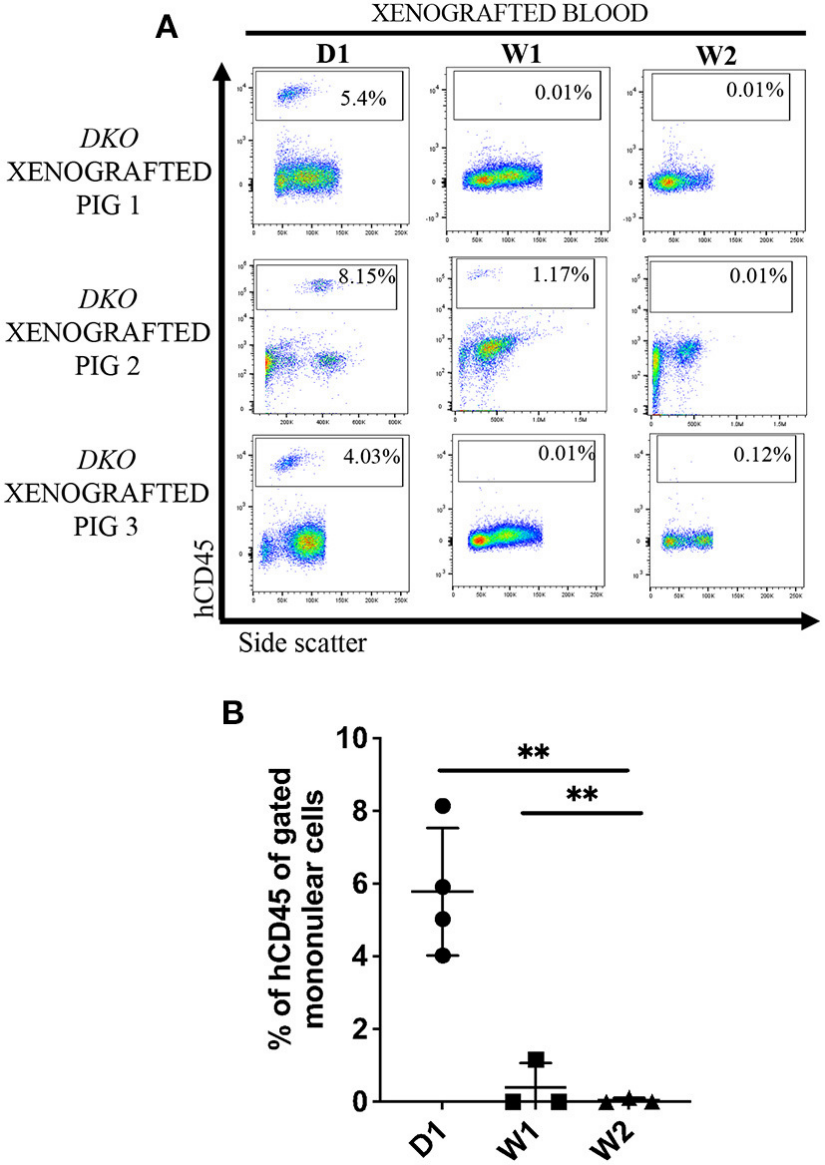
Postnatal clearance of human cells from the peripheral circulation in *DKO* xenografted pigs. **(A)** Flow cytometry analysis of peripheral blood and bone marrow for human CD45^+^ cells at birth (*n* = 1), 1 week (*n* = 1), and 2 weeks (*n* = 1) of age of three *DKO* xenografted pigs. While significant hCD45 cells were detectable at birth they reached nearly undetectable levels between 1 and 2 weeks of life. **(B)** Scatter plot showing the data presented in **(A)**. Data represent individual values for the percentage of human CD45 from mononuclear cells, line represents the mean, and adjusted *p* values are represented for Tukey's multiple comparisons tests one-way ANOVA with a significance value of 0.05. *P < 0.01.

Next, we set out to investigate the sustained presence of human lymphoid cells in peripheral blood of *DKO* pigs up to 3 weeks post birth. Peripheral blood from xenografted *DKO* pigs collected at 1 week (*n* = 3) and 2 weeks (*n* = 3) of age contained a detectable but decreasing frequency of human CD45^+^ cells (mean of 0.39% at 1 week and 0.12%, at 2 weeks) being nearly undetectable by 2 weeks of age (*p* = *0.0011)* (Figures 7A,B). These results suggest the presence of a post-natal barrier to persistence and/or expansion of human hematopoietic cells.

### Analysis of thymi from xenografted *DKO* pigs

**Figure 8:**
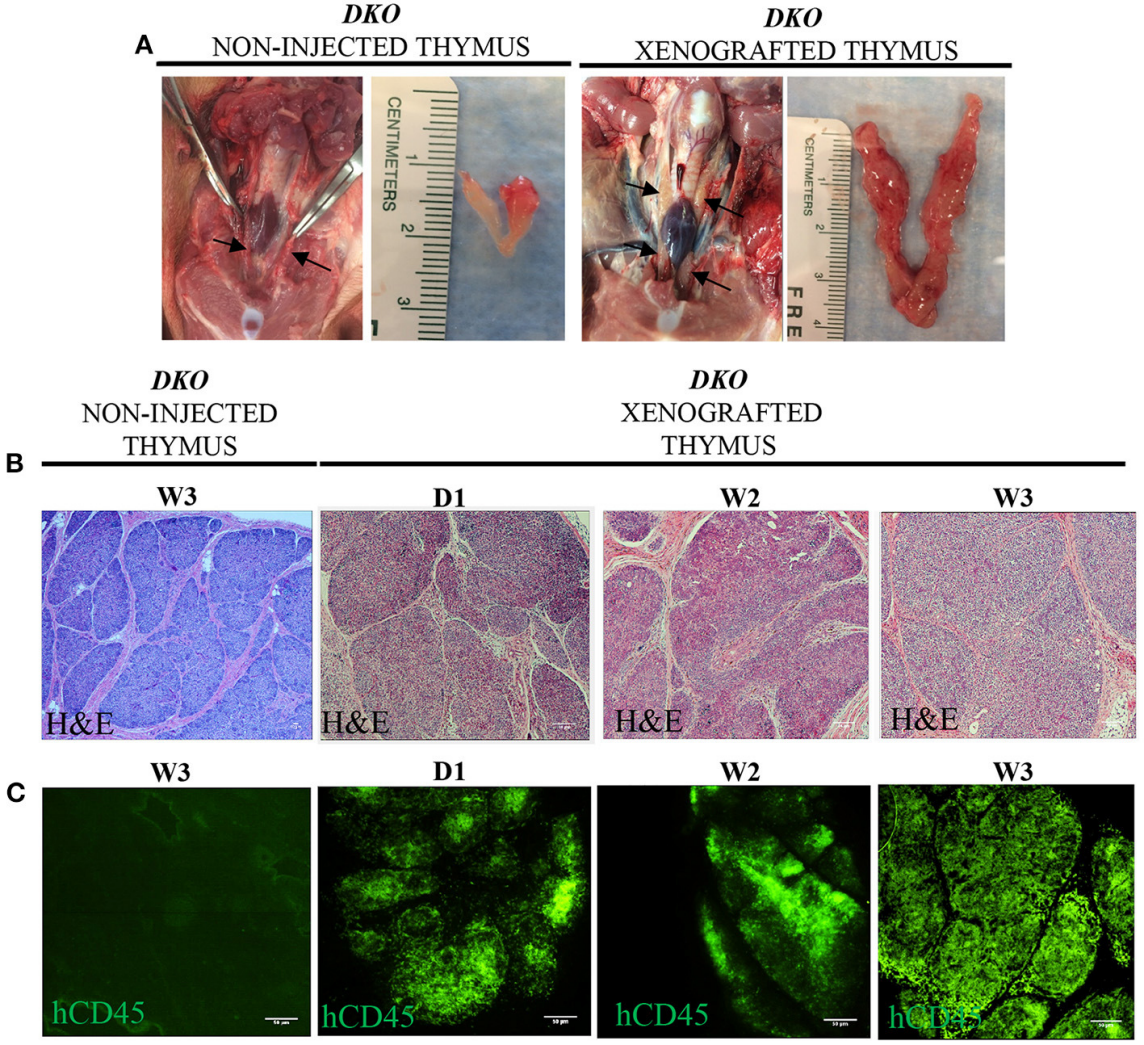
Macroscopic, microscopic and human CD45 immunofluorescence staining in the thymi of xenografted *DKO* pigs. **(A)** Representative macroscopic anatomy images of the thymus of non-injected and xenografted pigs. The xenografted thymus was noticeably expanded and larger than the non-injected thymus. **(B)** H&E histological structure (20x magnification) of thymi from non-injected pigs at 3 weeks and the thymi of three xenografted pigs at birth (*n* = 1), two (*n* = 1), and 3 weeks (*n* = 1) of age. **(C)** Immunofluorescence staining for human CD45^+^ cells with FITC conjugated antibodies is also shown. Non-injected thymus served as a negative control, showing no cross-reactivity.

**Figure 9:**
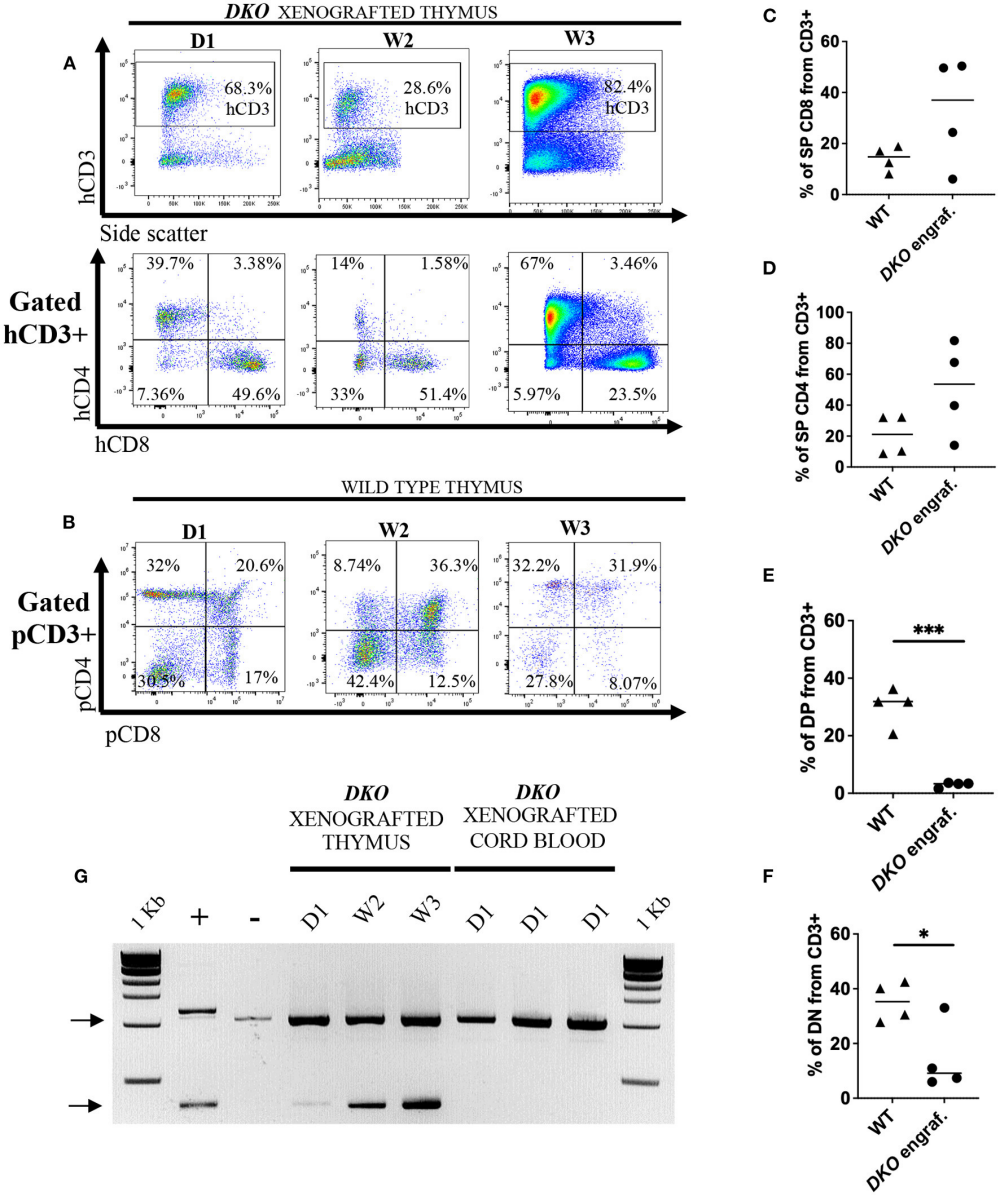
Sustained human engraftment of the thymus in *DKO* xenografted pigs. **(A)** Flow cytometry analysis from xenografted *DKO* pig thymi for hCD3, hCD4 and hCD8 from birth to 3 weeks of age. In contrast to peripheral blood and spleen, where human cells were undetectable by 2 weeks of age, in the thymus, robust engraftment could still be detected at 3 weeks of age. There were no changes in the distribution of SP hCD4, SP hCD8 with age. **(B)** Flow cytometry plots of wild-type pig thymi showing pig CD4 and pig CD8 expression in age-matched wild-type pig thymi. **(C–F)** Scatter plot with individual values for percentages (from CD3^+^) of pig (WT) and human (xenografted) CD3^+^ CD8^+^ **(C)**, CD3^+^ CD4^+^ **(D)**, DP **(E)** and DN **(F)** from wild type and xenografted thymi, respectively. Line represents the mean, and adjusted *p* values are represented for unpaired two-sample t-test with a significance value of 0.05. **(G)** PCR assay with human primers specific for identification of human TREC (~400 bp). Pig *IL2RG* amplicon served as internal primer control (~1 Kb) (Top black arrow). Each PCR reaction contains 50 ng of gDNA. Human peripheral blood mobilized stem cells gDNA served as positive control (+), while gDNA from the thymus of a non-injected *DKO* pig served as negative control (–). Samples tested include thymus from xenografted pigs (birth, 2 weeks, and 3 weeks), and gDNA from cord blood of xenografted *DKO* pigs at birth (when human cells were detected *via* flow cytometry).

Next, we examined the thymi of xenografted *DKO* pigs for the presence of human leukocytes. While histological analysis of xenografted *DKO* thymi showed poorly developed cortical zones and large medullary regions (Figures 8A,B), human CD45 staining confirmed the robust presence of human cells (Figure 8C). *T*-cell phenotype frequencies (hCD3, hCD4, hCD8, hDN and hDP) of xenografted thymi was compared to thymi of age-matched wild type pigs using pig specific antibodies (pCD3, pCD4, pCD8, pDN and pDP). Human CD3^+^ cells were detected at high frequencies at all-time points analyzed (Figure 9A). No differences were seen in frequencies of xenografted human hCD4 SP and hCD8 SP *T*-cells when compared to wild type pig CD4 SP (*p* = *0.11*) and pig CD8 SP cytotoxic *T*-cells (*p* = *0.22*) (Figures 9B–D). Xenografted pigs, however, displayed decreased frequencies of hDN *T*-cells compared to wild type pig DN *T*-cells (*p* = *0.02*) as well as decreased frequencies of hDP double positive *T*-cells compared to wild type pig DP double negative *T*-cells (*p* = *0.0002*) (Figures 9B,E,F).

To demonstrate that the thymi of xenografted *DKO* pigs can sustain human *T*-cell thymopoiesis, we examined for the presence of *T*-cell receptor excision circles (TREC) in the thymus at day 1 (*n* = 1), week 2 (*n* = 1), week 3 (*n* = 1), and cord blood (*n* = 3). Human TRECs were not detected in cord blood of xenografted *DKO* pigs (*n* = 3), even though these animals contained significant numbers of human *T*-cells at birth. However, human TRECs were detected in the thymus of xenografted *DKO* pigs at all stages examined.

### Spleen and bone marrow analysis of Xenografted *DKO* pigs

**Figure 10:**
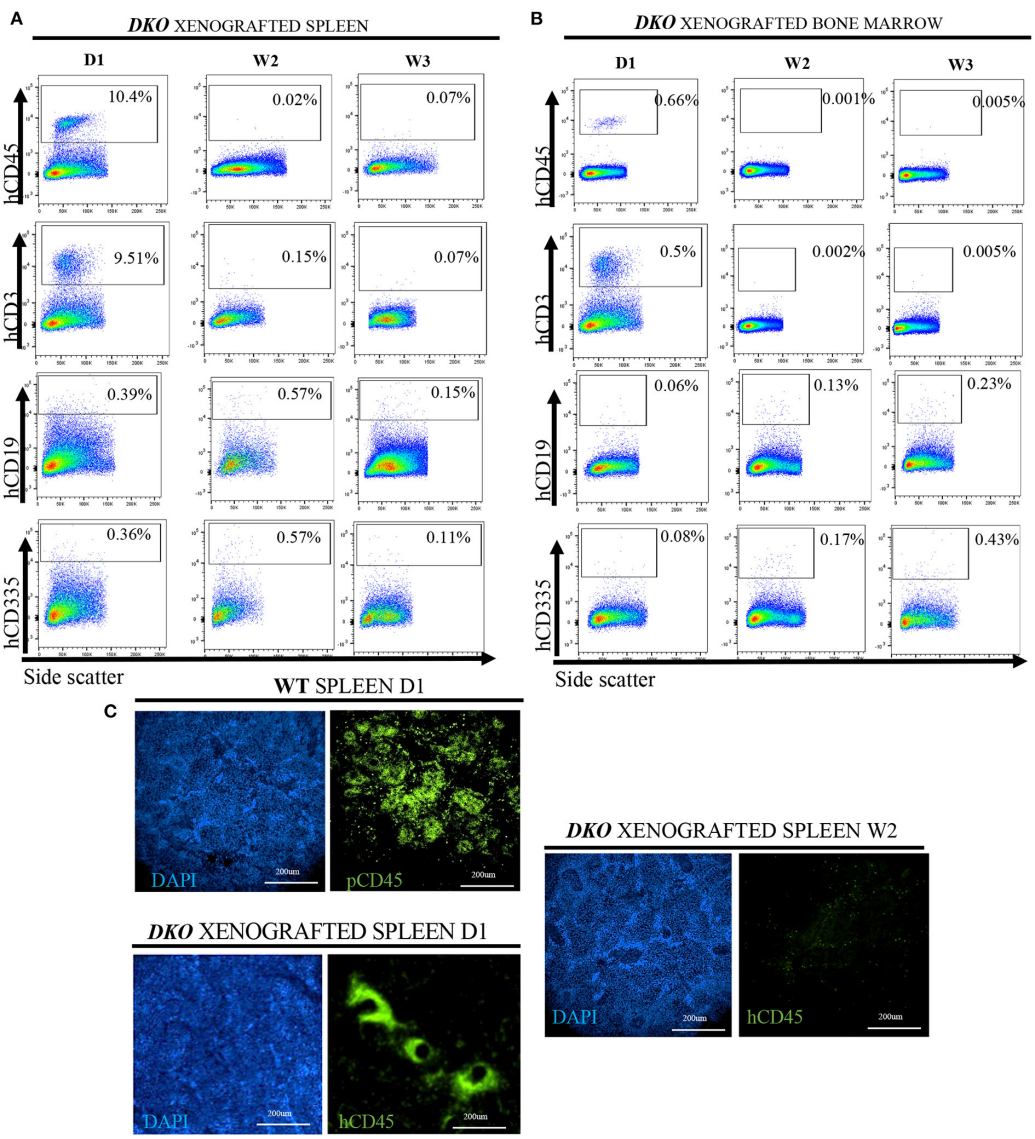
Postnatal clearance of human cells from the spleen and bone marrow of xenografted *DKO* pigs. Flow cytometry analysis for the presence of human CD45^+^, human CD3^+^, human CD19^+^ and human CD335 (versus side scatter) in the spleen **(A)** or bone marrow **(B)** of xenografted *DKO* pigs from birth to 3 weeks of age. Human CD45^+^ and human CD3^+^ cells were identified in the bone marrow and spleen of a *DKO* pig at birth but became undetectable between 2 and 3 weeks of age. Few, if any, hCD19 or hCD335 were detected at any stage of analysis. These data parallels that seen in circulating cells. **(C)** Detection and the overall organization of CD45+ cells in the spleen (20x magnification) of WT (pCD45) (*n* = 1) and xenografted *DKO* (hCD45) (*n* = 1) at day 1, and week 2 (*n* = 1). Human CD45^+^ cells were detected at day 1 but not at week 2 in *DKO* xenografted pigs, consistent with the clearance observed *via* flow cytometry. Immune-competent WT pig spleen staining with pig-specific CD45 antibodies showed multifocal distribution of pig CD45, as expected. In contrast, xenografted *DKO* day 1 spleen showed a different pattern, with human CD45 cells surrounding areas that resemble large vascular structures.

Since human *T*-cell development clearly occurred in the newborn thymus but human *T*-cells were scarce in the periphery of *DKO* pigs, we further asked whether xenograft persistence could be lymphoid tissue dependent. To answer this question, we studied the spleen and bone marrow of xenografted TKO for the presence of human CD45^+^, CD3^+^ (and co-expression of CD4 and CD8), human CD19^+^ and CD335^+^ cells. Human CD45^+^ and CD3^+^ cells were present in the spleen and bone marrow at birth, but human CD19^+^ and human CD335^+^ were nearly undetectable. Similarly, to peripheral blood analysis, the number of human *T*-cells in the spleen and bone marrow decreased after birth (Figures 10A,B). Lastly, we decided to demonstrate that human CD45^+^ cells were present in the spleen *via* immunofluorescence. As expected, human CD45^+^ cells were present in the spleen of xenografted *DKO* at day 1 but absent in the spleen of xenografted *DKO* at week 2. When compared to aged-matched WT spleen stained with pCD45, pCD45 staining showed an expected multifocal pattern resembling lymphoid structure. In contrast, hCD45 staining in the spleen of xenografted *DKO* pig at day 1 displays a different pattern, with human CD45 cells surrounding areas that resemble large vascular structures (Fiugre 10C). Taken together, these results suggest the presence of post-natal barriers present in lymphoid tissues (other than the thymus) impeding persistent survival of human cells in xenografted *DKO* pigs.

## Discussion

Human hematopoietic stem cells have been introduced into mature and fetal pigs, but engraftment in immunocompetent pigs has been so low that it could only be detected by PCR or by *in situ* detection of human Alu sequences (33). Previously we used *in utero* hematopoietic stem cell transplantation of human *T*-cell-depleted bone marrow and umbilical cord blood into wild type pig fetuses, leading to low human *T*-cells present in peripheral blood with a marked decline in chimerism within 1 week from birth (33). One report showed presence of human cells using flow cytometric analysis (36) and identified human cells in the thymus (1.6% hCD45) and bone marrow (1.1% hCD45) of a fetus injected with human CD34^+^ cells at D35 of gestation and collected at D90 of gestation. Peripheral blood levels of human CD45 at birth were 0.58% and there was no evidence of B cell or NK cells. Collectively these reports demonstrate that, while immunocompetent pigs can sustain human lymphoid lineage engraftment after *in utero* hematopoietic stem cell transplantation, the level of engraftment is too low to be of practical use. Suzuki et al. (15) described successful allogeneic postnatal bone marrow transplantation of *IL2RG* knock-out pigs. In this study, successful engraftment was established with and without conditioning, resulting in variable T, NK and B cell engraftment, and consequently, increased lifespan of certain animals when compared to non-injected controls.

A separate study continues the steps of developing immunodeficient pigs for allogeneic and xenogeneic transplantation studies. Lee et al. (17) generated and characterized a *RAG2* deficient transgenic pig model, and demonstrated successful engraftment of human iPSC and allogeneic trophoblast stem cells. A *DKO* pig model has been described by Lei et al. (20) using direct injection of CRISPR/Cas9 system in developing embryos; no stem transplantation studies were performed. Importantly the immunodeficient phenotype or the *DKO* model described here is consistent with the results of Lei et al. (20) showing marked reduction in peripheral blood B, T and NK cells, while also showing poor lymphoid tissue development. To our knowledge, this is the first report carrying out allogeneic and xenogeneic stem cells *in utero* transplantation in a IL2RG/RAG2 *DKO* pig model.

Using gene-edited pigs lacking *IL2RG* and *ART*, Boettcher et al. (22) demonstrated successful establishment of an immunodeficient phenotype, with main lymphoid cells nearly undetectable in peripheral blood and lymphoid tissue, resembling the model reported by Lei et al. (20) and our group. Boettcher et al. (22) further carried out post-natal allogeneic SLA matched bone marrow transplantation, as well as *in utero* hematopoietic stem cell xenotransplantation. Using SLA matched bone marrow (female donor, 4y) and intravenous infusion into a 5 days old male *IL2RG*/*ART* null piglet, they reported successful allogeneic engraftment of T and NK cells but limited to no B cell engraftment. Overall chimerism was monitored and maintained for at least 4 months. Major differences with the present hematopoietic allogeneic transplantation study include (a) *DKO* vs. *ART* ^−/−^*IL2RG* ^*y*/−^; (b) SLA mismatched vs. SLA matched; and (c) postnatal bone marrow donor HSC vs. fetal liver HSCs.

In the *DKO* background, our results show sustained allogeneic engraftment of SLA mismatched pH2B-eGFP-tagged fetal liver mononuclear cells containing hematopoietic stem cells. There are seven classical class I genes and three non-classical class I genes mapped to the SLA complex. The constitutively expressed classical *SLA* class I genes are *SLA-1, SLA-2* and *SLA-3*, while the rest are pseudogenes. SLA class II loci in the pig include *DRB1, DQA, DQB1, DOB1, DMB, DMA, and DOA* (28). In humans, histocompatibility testing between unrelated donors and recipients prior to hematopoietic stem cell transplantation is performed for five loci: *HLA-A, -B, -C, -DRB1*, and *DQB1*. Optimal transplantations are defined as 10/10, a perfect match between the critical 5 *HLA* locus cited above between donor and recipient. 8/8 refers to high-resolution matching at four of the loci. Minimum requirements for adult bone marrow and peripheral blood mobilized cells transplantation are 6 of 8 loci matches for *HLA-A, -B, -C* and *DRB1* (37). In addition, the presence of donor antigens or alleles not shared by the recipient determines host vs. graft allorecognition, while the presence of recipient alleles or antigens not shared by the donor determines graft vs. host allorecognition, both individual scenarios are classified as unidirectional mismatch and may lead to failure of engraftment (38). In addition, both scenarios may occur simultaneously between a single donor and recipient (each one having an allele that the other one does not have), leading to a bidirectional mismatch and failure of engraftment (38). All three cell donors were classified as poor donors, since a perfect match was not observed for SLA-1, 2, 3 and DRB1 and DQB1. The lack of B cell, CD8 SP and DP cells in *DKO* pigs allowed substantial engraftment of these lineages (Figures 3, 4). Variable mix of host and donor cells was observed within the CD4 SP cell population (Figure 3F). We further demonstrated that rearranged *IgH* and *TCR*-β could be detected post transplantation in the spleen and thymus of allografted pigs, respectively. Taken together, these data reflect the immunodeficient phenotype of the *DKO* pig model, and its ability to engraft with SLA-mismatched allogeneic cells, while also highlighting the utility of the pH2B-eGFP model. Further, an in-utero stem cell transplantation approach may take advantage of the tolerant immunologically privileged status of the fetus and represents an alternative large animal model for *in utero* clinical therapies of immunodeficiency disorders (39). It is also important to note the limitations of our allogeneic studies as due to technical difficulties, we were not able to investigate allogeneic engraftment in peripheral blood nor did we examine myeloid or sustained long-term engraftment.

Notable, similar to our transplantation study, Boettcehr et al. (22) also reported *in utero* cord blood derived human CD34^+^ stem cell transplantation in *IL2RG* ^−/−^*ART* ^−/−^ fetuses. They reported a total of three piglets, one euthanized at birth and two euthanized at day 1, with only two showing any significant engraftment and the third low to no engraftment (<1%). Thus, their results are based on two observations. Their approach differs not only in the mutant genetic background used but also on the source of human hematopoietic cells. While we used CD34^+^ enriched from peripheral blood of normal donors, Boettcher et al. (22) used in vitro cultured and expanded CD34 cells. They also report a smaller number of xenografted animals (2 vs. 4) and the longer period the examined engraftment was one day vs. 3 weeks in this report. However, similar to our results, they reported that the two engrafted piglets displayed evidence of human CD45^+^ cell engraftment in peripheral blood and lymphoid organs. Human CD45^+^ cells were predominantly CD3^+^ cells, with one animal displaying human *T*-cell levels of 63% in the thymus.

Using the *DKO* mutant genetic background, we confirm and extend these observations and demonstrate that *in utero* hematopoietic stem cell transplantation of peripheral blood mobilized human CD34^+^ cells into *DKO* pig fetuses result in never before reported levels of engraftment ranging from 80% at week 3 (thymus) (Figure 9) to 8% (cord blood) (Figures 5, 6). To our knowledge, this is the highest human hematopoietic engraftment achieved in a pig model. While significant engraftment with human cells was detected, the pattern of engraftment varied from that seen in an immune-competent human or wild-type pigs. In the xenografted pigs, T-cells were identified in blood, spleen and thymus at birth. In contrast, B and NK cell production was minimal, if any. In addition, examination of CD4 and CD8 populations revealed a shift when compared with human or immunocompetent pigs. As shown in Figures 5, 6, there was a significant increase in peripheral blood DN CD4 CD8 cells in the xenografted animals compared to normal human peripheral blood. In the thymus, in contrast, the opposite was true, with the xenografted thymus having a low population of DN and DP *T*-cells compared to wild type thymus (Figure 9). This could reflect hastened maturation or uncoordinated early development.

TRECs are generated during V(D)J gene recombination, a process responsible for the diversity of *T*-cell antigen receptor (TCR) repertoire. This complex end-to-end fusion of gene segments is mediated by recombination-activating genes *RAG1/2* that recognize “heptamer–spacer–nonamer” recombination signal sequences (RSSs) flanking each V, D and J gene segment. Generation of a coding TCR chain results in the excision of extrachromosomal DNA circles (TREC). Therefore, its identification correlates with *T*-cell differentiation (33, 40). We selected previously reported human-specific primers capable of detecting *TCR*δ to *TCR*α recombination (δ*Rec-*Ψ*J*α), since δ*Rec-*Ψ*J*α represents ~67% of recombination events (41, 42). All the thymi from xenografted animals showed the presence of TRECs. As duplication of TREC circles within replicating mature *T*-cells does not occur (43), the presence of human TREC suggests the pig thymus can sustain de novo human *T*-cell thymopoiesis. Together these results indicate that while the thymus of *DKO* pigs accept *T*-cell precursor cells and support development of phenotypically mature *T*-cells, mature *T*-cells are either not released from the thymus or are released but rapidly cleared in the periphery. Further, by contrasting the results to peripheral blood human leukocyte clearance, it may suggest that the *DKO* thymus is an immunological safe niche for the presence of human leukocytes.

It should be noted that the xenografted profile of *DKO* pig differs considerably from the *RAG2*^−/−^*Il2RG*^*y*/−^ mouse. While in the *DKO* pig, the engraftment is essentially T-cell driven in all tissues examined, in mice, B-cells predominate over T-cells (2-3X higher), and in the thymus, the T- cell population is dominated by double positive cells (>70%). This is drastically different in the *DKO* pig with SP CD4 and SP CD8 being the two main T-cell populations seen in thymus at all stages examined. Although the thymi of xenografted *DKO* pigs displayed lower frequencies of DP and DN *T*-cells when compared to age matched wild type pig thymus, detection of human TREC is suggestive that human *T*-cell development can proceed to the generation of human T-cells (or at least initiate to that stage). The mouse thymus does not support maturation of human T-cells and requires the presence of human or pig fetal thymic tissue (44, 45). Our observations confirm human T-cell development in a pig thymus (45).

Also, the xenogeneic transfers used enriched CD34 cells and allogeneic transfers utilized fetal liver cells as a source of hematopoietic stem cells as reliable pig CD34 antibodies are not available. Notwithstanding this donor source difference, the data shows that allogeneic CD34^+^ can generate all lymphoid lineages while xenogeneic HSC can only generate T-cells. Thus, the lack of B-cells in the *DKO* pig suggests that the cytokines responsible for controlling proliferation and/or differentiation of CD34^+^ HSC into B or NK cells are incompatible between pigs and humans. In mice, some of these deficiencies have been overcome by the humanized MISTRG mice that express the human factors M-CSF, IL-3, SIRP-a, GM-CSF and TPO (46). This strain yields significantly higher engraftment of human bone marrow cells and produces more functional NK cells, T and B cells. We feel that a similar approach will also improve the development of B and NK cells in the *DKO* pig. It is also important to note the limitations of our xenogeneic studies, as our study did not examined for the presence of human myeloid and RBC cells, human bone marrow CD34^+^ in pigs bone marrow, testing of different sources of human CD34^+^ cells (cord blood, bone marrow), or presence of human T regulatory cells in the thymi of xenografted pigs.

An additional issue in the *DKO* pig is the rapid clearance of human cells from peripheral blood and spleen soon after birth, suggesting non-lymphoid barriers to human engraftment. The one exception was the thymus capable of sustaining never reported before levels of human CD3 engraftment (up to 82.4% at 3 weeks). The reason for this clearance remains unexplained but could be due to incompatibilities between SIRP-α and CD47. Incompatibility between CD47 and SIRP-a results in phagocytosis of donor cells by host macrophages. Expression of human SIRP-α in immunodeficient mice abolishes this incompatibility and results in higher human cell engraftment and mobilization (47). In addition, it has been shown that pig CD47 does not interact with human SIRP-α (48) and others have shown that expression of human CD47 in pig cells increases engraftment in a mouse model of pig-to-human transplantation (49). Boettcher et al. (50), however, has shown that there is at least partial binding between the two molecules with an increase in phagocytosis on human cells by pig monocytes from 5% in controls to 20% when using antibodies that block the CD47 and SIRP-α interaction. We are now examining this in more detail to determine the role of CD47-SIRP-α incompatibility in the rapid clearance of human cells in the xenografted *DKO* pigs.

In conclusion, while hematopoietic engraftment in an immunodeficient pig has been reported before using a *IL2RG/ART* null pig (22), this is, to our knowledge, the first report that demonstrates prolonged engraftment (3 weeks vs. one day), rapid clearance of human cells postnatally, and the presence of human TRECs supporting de novo T- cell development in the thymus. In addition, we present novel data not previously reported in either allogeneic or xenogeneic transplantation in a *RAG/IL2RG* null (*DKO*) pig. This model can be used for understanding the limitations of *in utero* hematopoietic stem cell transplantation of human CD34^+^ cell engraftment, and also as a first step toward improving transplantation of human stem cells from different tissues/sources for generation of chimeric human tissues and organs for xenotransplantation applications in a large animal model.

## Data availability statement

The original contributions presented in the study are included in the article/Supplementary material, further inquiries can be directed to the corresponding author.

## Ethics statement

This study was carried out in strict accordance with the recommendations in the Guide for the Care and Use of Laboratory Animals of the National Institutes of Health. The animals used in this study were obtained from a university-owned herd, and all animal procedures were approved by the Institutional Animal Care and Use Committee of North Carolina State University (Raleigh, NC).

## Author contributions

RS and JAP: conception and design, acquisition of data, analysis and interpretation of data, and drafting of manuscript. OL, JP, TK, and KP: conception and design, acquisition of data, analysis, and interpretation of data. LG: conception and design and acquisition of data. LB: analysis and interpretation of data. SS, KG, XZ, BC, YM, and JLP: acquisition of data. JLP: conception and design, interpretation of data, and drafting of manuscript. All authors contributed to the article and approved the submitted version.

## Funding

Funding provided by NIH grants R01-OD023138 and R01HL051587 to JAP and JLP.

## Conflict of interest

The authors declare that the research was conducted in the absence of any commercial or financial relationships that could be construed as a potential conflict of interest.

## Publisher's note

All claims expressed in this article are solely those of the authors and do not necessarily represent those of their affiliated organizations, or those of the publisher, the editors and the reviewers. Any product that may be evaluated in this article, or claim that may be made by its manufacturer, is not guaranteed or endorsed by the publisher.

## Supplementary material

Supplementary Figure 1 Enrichment systems for *IL2RG* and *RAG2* genome targeting in somatic cell porcine fetal fibroblast. **(A)** Schematic representation of nuclease reported vectors (pSSA) for modification of *IL2RG*. *IL2RG* TALEN reported vector contains GFP gene split into two inactive fragments containing overlapping homologies with the TALEN site between them flanked by *NheI* and *EcoRI* restriction sites. In cells, cleavage at the TALEN site will initiate single strand annealing and generate an active GFP gene. **(B)** Fluorescence activated cell sorting analysis to enrich for *IL2RG* edited fetal fibroblast lines. Negative controls group include no vector (–) and pSSA only, while pMAX served as GFP control (+), GFP+ cells from pSSA + *IL2RG* TALEN group (4.2%) were sorted for generation of single cell colonies. *IL2RG*^−/*y*^ cells were screened and selected for a second round of somatic cell nuclear transfer, followed by fetal fibroblast isolation and subsequent *RAG2* editing. **(C)** Schematic representation of reporter plasmid used for enrichment of *RAG2* modification, containing CMV promoter, RPF, *RAG2* CRISPR-Cas9 gRNA target binding site (flanked by *EcoRI* and *BamHI*) followed by 2A peptide and hygromycin fused with GFP. Cells were treated with 2 mg/ml hygromycin for 48 h, 48 h post-transfection. **(D)** Schematic representation of the *RAG2* pig locus, the red line indicates the CRISPR-Cas9 binding site, arrows indicate primers used for PCR, the amplicon contains 3 *HpyCH4V* restriction sites used for screening. **(E)** Fluorescence microscopy of *IL2RG*^−/*y*^ cell line 48 h post-transfection showing reported alone transfected cells to only express RFP (texas red channel) (transfection control), while few cells transfected with *RAG2* CRISPR-Cas9 and reporter co-expresses RFP and GFP, indicating reporter activity. **(F)** Single cell colony PCR and *HpyCH4V* assay for detection *DKO* cells lines. Wild type (WT) PCR amplicons digested and undigested with *HpyCH4V* served as controls. The presence of three bands indicates heterozygous lines for *RAG2*, while the presence of two bands in conjunction with the disappearance of the 190 bp band indicates homozygous lines. Colonies selected for sequencing are indicated. **(G)** Overall targeting efficiency of the pig *RAG2* locus, indicating total indels frequency (79%), heterozygous (51%) and homozygous (28%).

Supplementary Figure 2 Haplotype characterization of pH2B-eGFP pig donors and DKO line, and human hematopoietic stem cell selection and sex determination. **(A)** Haplotype PCR gels for *DKO* line, and pH2B-eGFP fetal liver donors 1, 2, and 3 used for *in utero* allogeneic transplants. Red boxes indicate the MHC-I (SLA-1, SLA-2, SLA-3) and MHC-II (DRB1, DQB-1, and DQA) PCR reactions with different primers. A Positive internal control primer (porcine a-actin gene, 516 bp) is present in all reactions. Each well also contains 1 pair of primers specific for the multicopy alleles for each sub-haplotype group. The presence of a smaller band indicates a positive signal for the corresponding allele. **(B)** Summary of the findings obtained by PCR. Donors were found to be mostly different MHC-I and MHC-II overall haplotypes. **(C)** Flow cytometry analysis of donor A CD34 + cells utilized for *in utero* xenogeneic transplants. Peripheral blood mobilized stem cells were stained with anti-human CD34 antibodies prior to magnetic isolation (pre CD34 enrichment) and after purification in magnetic columns (post CD34 enrichment), revealing a 97.7% enrichment of CD34+ cells. **(D)** PCR assay for detection of X and Y chromosomes from donor A and donor B human cells. A set of primers were used to amplify the human X and Y chromosome, with male (M) and female (Y) templates serving as gender controls. Donor cells were found + for X and Y chromosomes, revealing donor A and B be of the male sex.

Supplementary Figure 3 Timeline schematic of all *in vivo* procedures and sample harvests performed.

Supplementary Figure 4 DNA sequence of single cell colonies generated by nucleofection of *IL2RG* targeting TALENs and *RAG2* targeting CRISPR-Cas9. DNA sequencing of target region detected from selected single cell *IL2RG*^−/*y*^ (TALEN) colonies. Also shown is a list of *RAG2* mutations (homozygous and heterozygous) identified in *DKO* cell lines (generated on the *IL2RG* background). TALEN binding sites are represented for bold and underline sequences, while bold sequences represent PAM sequence. Allelic status is also shown.

Supplementary Figure 5 Evidence of loss of rearranged *TCR-*β locus in the thymus and *IgH* locus in the spleen of *DKO* pigs. **(A)** Detection of *D-J TCR-*β locus rearrangement in thymus gDNA. A germinal band is present in all samples (~1,500 bp), while the presence of bands smaller than 750 bp (indicated by black arrow) indicates rearranged *TCR-*β locus. This band is present in the thymus of wild-type pigs and absent in the thymus of *DKO* pigs. **(B)** Detection of V(D)J *IgH* locus rearrangement in spleen gDNA. A germinal band is present in all samples (~1,500 bp), while the presence of a 500 bp (indicated by black arrow) indicates rearranged *IgH* locus. This band is present in the spleen of wild-type and absent in the spleen of *DKO* pigs. This indicates the lack of *RAG2* activity.
