# Supplementary figures and images for: Allogeneic and xenogeneic lymphoid reconstitution in a RAG2−/−IL2RGy/− severe combined immunodeficient pig: A preclinical model for intrauterine hematopoietic transplantation
Source: Front Vet Sci. 2022 Oct 14;9:965316. doi: 10.3389/fvets.2022.965316 (PMC9614384; doi:10.3389/fvets.2022.965316)

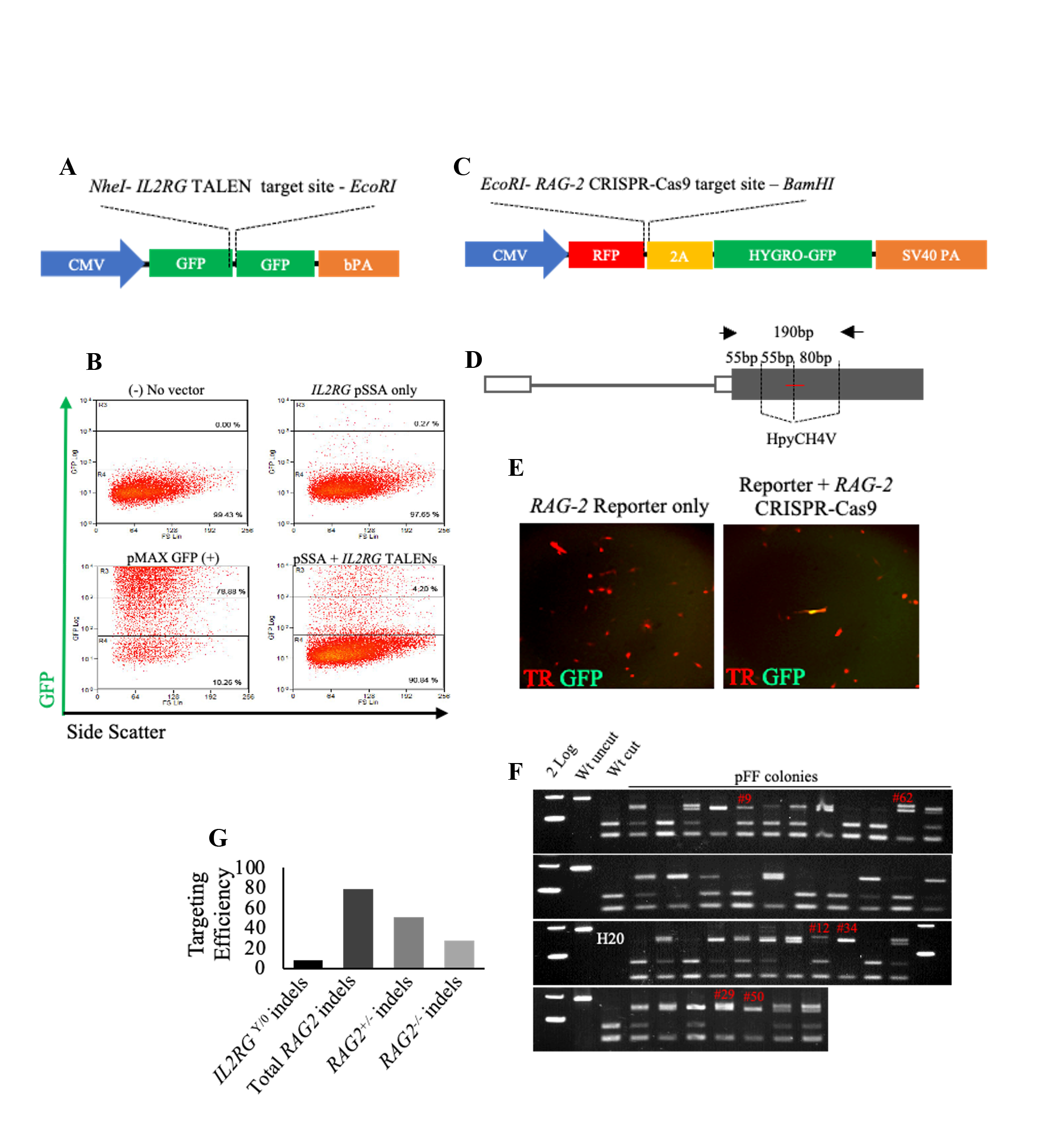

Supplement: Supplementary Figure 1 — Enrichment systems for IL2RG and RAG2 genome targeting in somatic cell porcine fetal fibroblast. (A) Schematic representation of nuclease reported vectors (pSSA) for modification of IL2RG. IL2RG TALEN reported vector contains GFP gene split into two inactive fragments containing overlapping homologies with the TALEN site between them flanked by NheI and EcoRI restriction sites. In cells, cleavage at the TALEN site will initiate single strand annealing and generate an active GFP gene. (B) Fluorescence activated cell sorting analysis to enrich for IL2RG edited fetal fibroblast lines. Negative controls group include no vector (–) and pSSA only, while pMAX served as GFP control (+), GFP+ cells from pSSA + IL2RG TALEN group (4.2%) were sorted for generation of single cell colonies. IL2RG−/y cells were screened and selected for a second round of somatic cell nuclear transfer, followed by fetal fibroblast isolation and subsequent RAG2 editing. (C) Schematic representation of reporter plasmid used for enrichment of RAG2 modification, containing CMV promoter, RPF, RAG2 CRISPR-Cas9 gRNA target binding site (flanked by EcoRI and BamHI) followed by 2A peptide and hygromycin fused with GFP. Cells were treated with 2 mg/ml hygromycin for 48 h, 48 h post-transfection. (D) Schematic representation of the RAG2 pig locus, the red line indicates the CRISPR-Cas9 binding site, arrows indicate primers used for PCR, the amplicon contains 3 HpyCH4V restriction sites used for screening. (E) Fluorescence microscopy of IL2RG−/y cell line 48 h post-transfection showing reported alone transfected cells to only express RFP (texas red channel) (transfection control), while few cells transfected with RAG2 CRISPR-Cas9 and reporter co-expresses RFP and GFP, indicating reporter activity. (F) Single cell colony PCR and HpyCH4V assay for detection DKO cells lines. Wild type (WT) PCR amplicons digested and undigested with HpyCH4V served as controls. The presence of three bands indicates heterozygous lin [file Image_1.TIF]

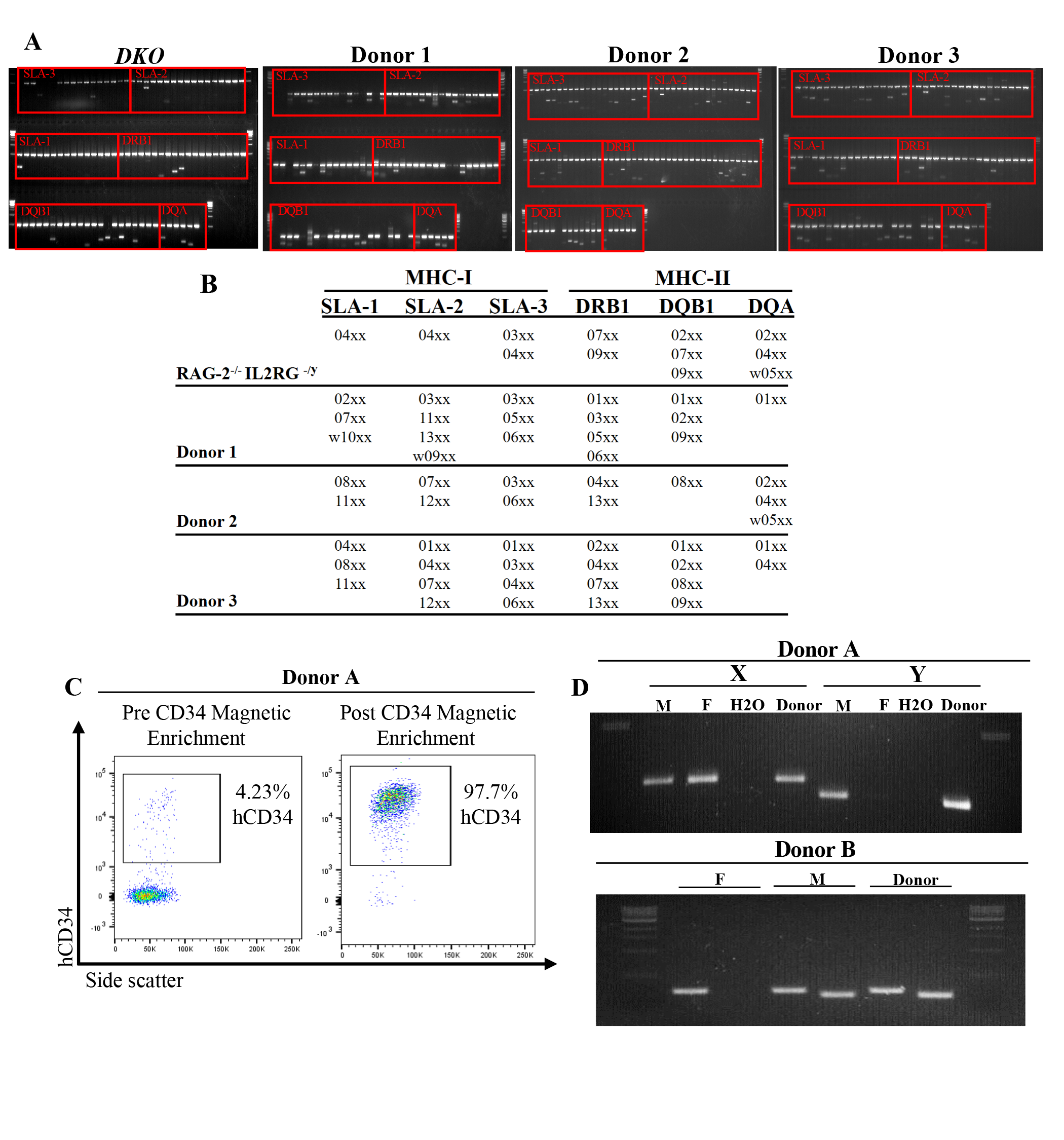

Supplement: Supplementary Figure 2 — Haplotype characterization of pH2B-eGFP pig donors and DKO line, and human hematopoietic stem cell selection and sex determination. (A) Haplotype PCR gels for DKO line, and pH2B-eGFP fetal liver donors 1, 2, and 3 used for in utero allogeneic transplants. Red boxes indicate the MHC-I (SLA-1, SLA-2, SLA-3) and MHC-II (DRB1, DQB-1, and DQA) PCR reactions with different primers. A Positive internal control primer (porcine a-actin gene, 516 bp) is present in all reactions. Each well also contains 1 pair of primers specific for the multicopy alleles for each sub-haplotype group. The presence of a smaller band indicates a positive signal for the corresponding allele. (B) Summary of the findings obtained by PCR. Donors were found to be mostly different MHC-I and MHC-II overall haplotypes. (C) Flow cytometry analysis of donor A CD34 + cells utilized for in utero xenogeneic transplants. Peripheral blood mobilized stem cells were stained with anti-human CD34 antibodies prior to magnetic isolation (pre CD34 enrichment) and after purification in magnetic columns (post CD34 enrichment), revealing a 97.7% enrichment of CD34+ cells. (D) PCR assay for detection of X and Y chromosomes from donor A and donor B human cells. A set of primers were used to amplify the human X and Y chromosome, with male (M) and female (Y) templates serving as gender controls. Donor cells were found + for X and Y chromosomes, revealing donor A and B be of the male sex. [file Image_2.TIF]

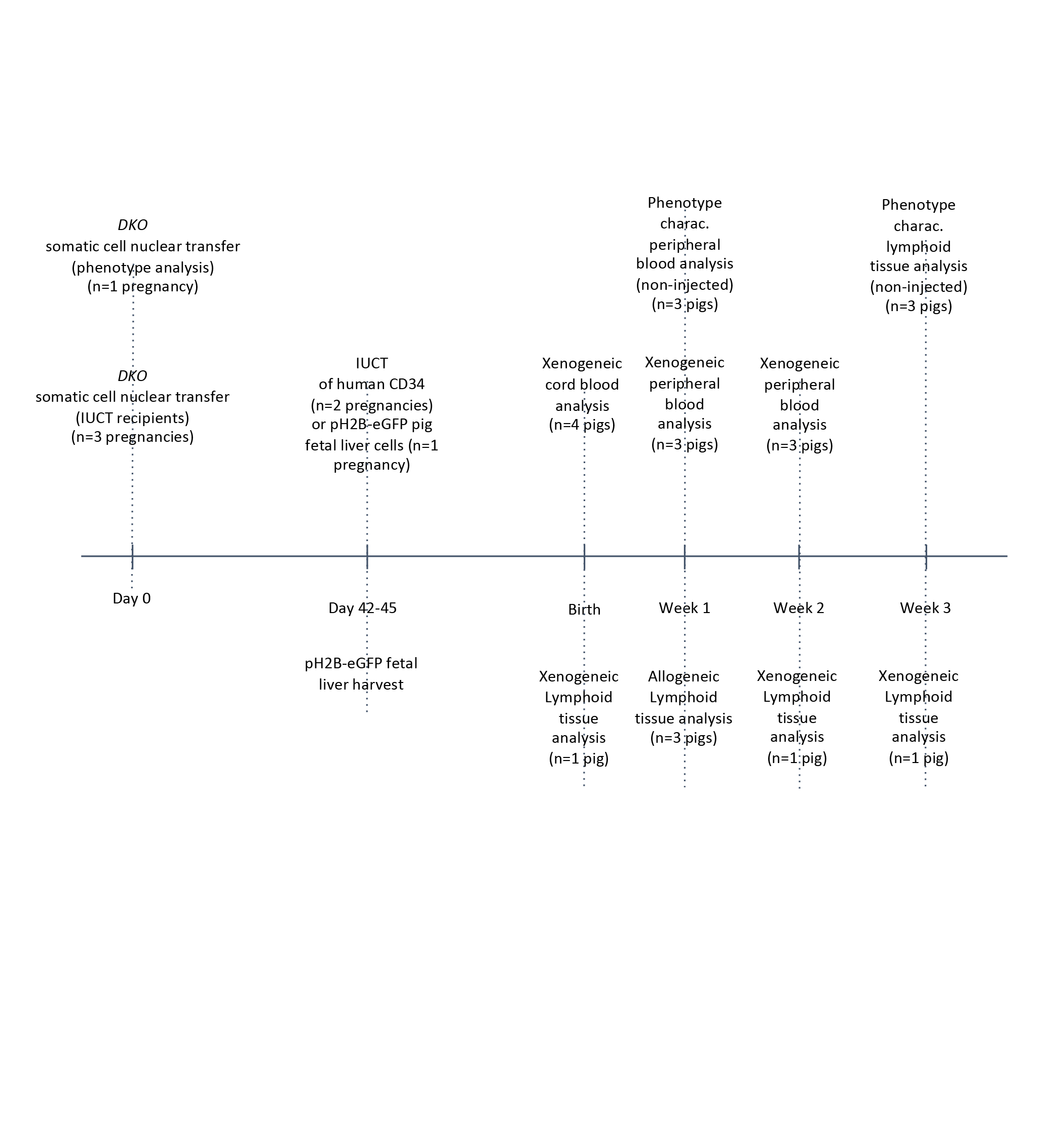

Supplement: Supplementary Figure 3 — Timeline schematic of all in vivo procedures and sample harvests performed. [file Image_3.TIF]

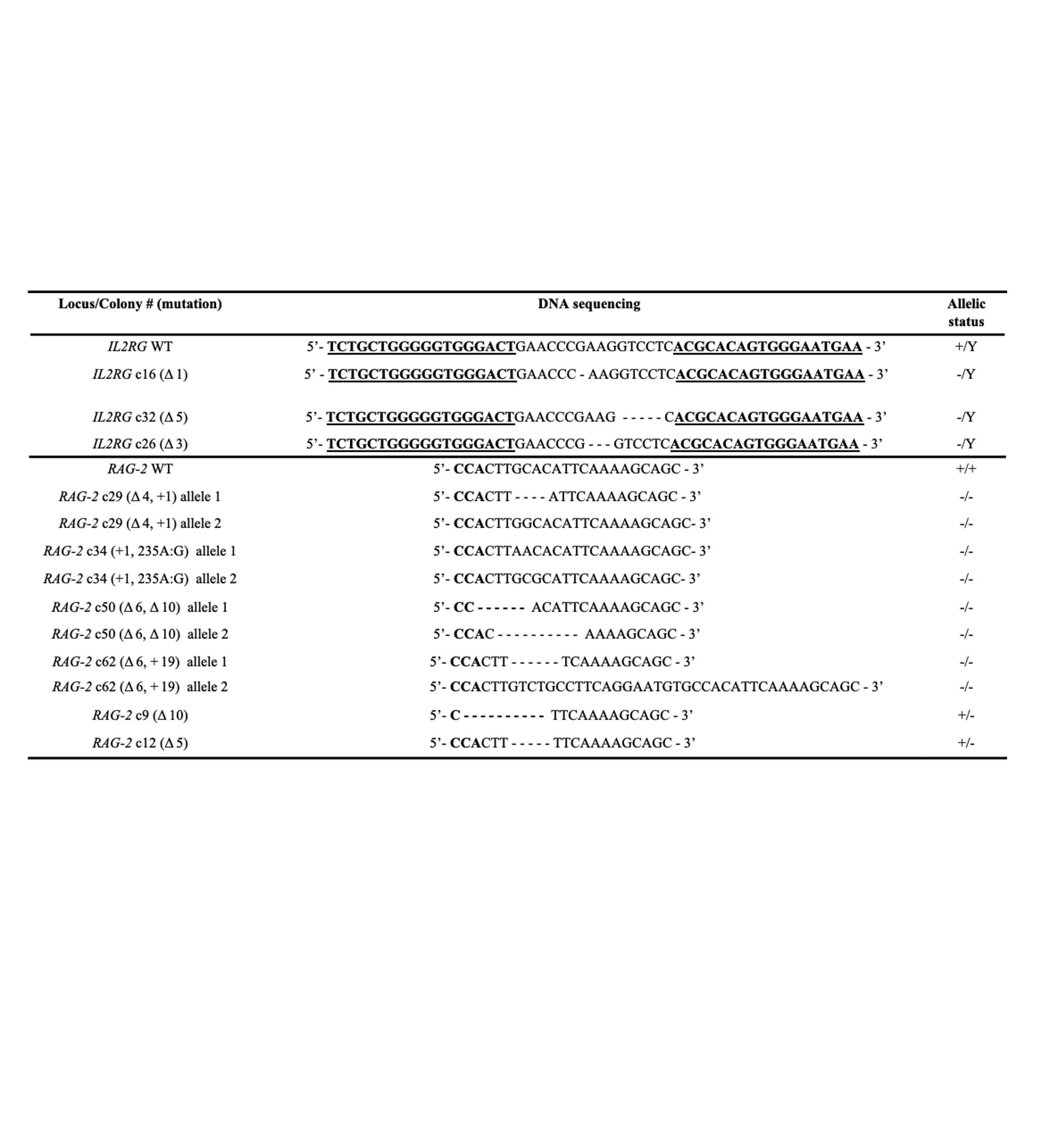

Supplement: Supplementary Figure 4 — DNA sequence of single cell colonies generated by nucleofection of IL2RG targeting TALENs and RAG2 targeting CRISPR-Cas9. DNA sequencing of target region detected from selected single cell IL2RG−/y (TALEN) colonies. Also shown is a list of RAG2 mutations (homozygous and heterozygous) identified in DKO cell lines (generated on the IL2RG background). TALEN binding sites are represented for bold and underline sequences, while bold sequences represent PAM sequence. Allelic status is also shown. [file Image_4.TIF]

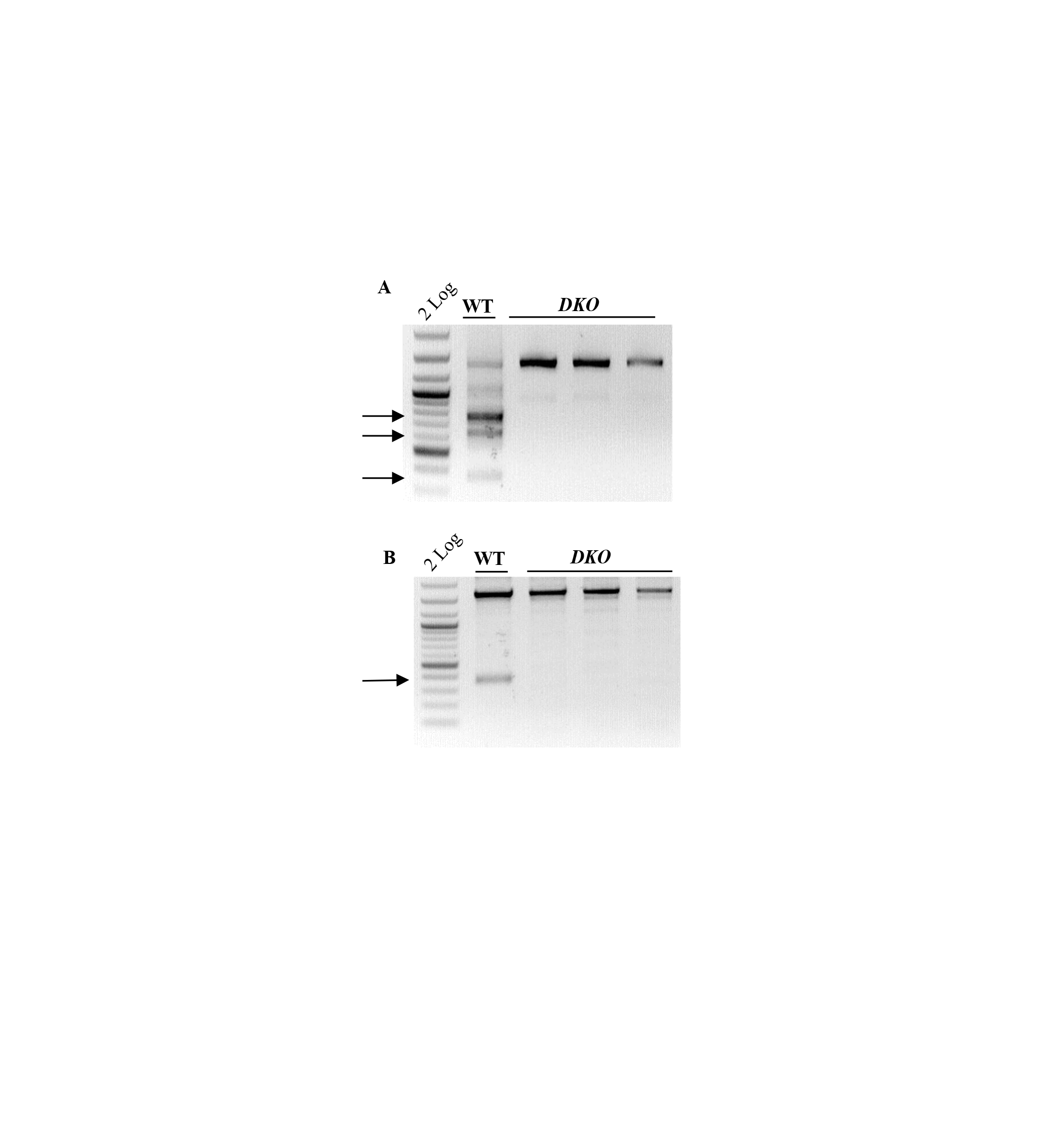

Supplement: Supplementary Figure 5 — Evidence of loss of rearranged TCR-β locus in the thymus and IgH locus in the spleen of DKO pigs. (A) Detection of D-J TCR-β locus rearrangement in thymus gDNA. A germinal band is present in all samples (~1,500 bp), while the presence of bands smaller than 750 bp (indicated by black arrow) indicates rearranged TCR-β locus. This band is present in the thymus of wild-type pigs and absent in the thymus of DKO pigs. (B) Detection of V(D)J IgH locus rearrangement in spleen gDNA. A germinal band is present in all samples (~1,500 bp), while the presence of a 500 bp (indicated by black arrow) indicates rearranged IgH locus. This band is present in the spleen of wild-type and absent in the spleen of DKO pigs. This indicates the lack of RAG2 activity. [file Image_5.TIF]
